# Essential genes of the macrophage response to *Staphylococcus aureus* exposure

**DOI:** 10.1186/s11658-018-0090-4

**Published:** 2018-05-23

**Authors:** Aixia Sun, Hongwei Zhang, Feng Pang, Guifen Niu, Jianzhong Chen, Fei Chen, Jian Zhang

**Affiliations:** 1 Department of Clinical Laboratory, Liaocheng People’s Hospital, 67 West Dongchang Road, Liaocheng, 252000 Shandong Province People’s Republic of China; 2 Department of Endocrinology, Liaocheng People’s Hospital, 67 West Dongchang Road, Liaocheng, 252000 Shandong Province People’s Republic of China; 3 Department of Clinical Pharmacy, Liaocheng People’s Hospital, 67 West Dongchang Road, Liaocheng, 252000 Shandong Province People’s Republic of China; 4 Outpatient Vaccination Service, Center for Disease Control and Prevention of Liaocheng, Liaocheng, 252000 Shandong Province People’s Republic of China

**Keywords:** Macrophage, *Staphylococcus aureus*, Bio-informatics

## Abstract

**Background:**

Although significant advances have been made in understanding the mechanisms of macrophage response to *Staphylococcus aureus* infection, the molecular details are still elusive. Identification of the essential genes and biological processes of macrophages that are specifically changed at different durations of *S. aureus* exposure is of great clinical significance.

**Methods:**

We aimed to identify the significantly changed genes and biological processes of *S. aureus*-exposed macrophages. We systematically analyzed the macrophage gene expression profile GSE 13670 database with 8 h, 24 h or 48 h *S. aureus* infection. The results were further confirmed by western blot and quantitative polymerase chain reaction (qPCR) analyses.

**Results:**

After 8 h of *S. aureus* infection, the expression of 624 genes was significantly changed. Six hundred thirteen differentially expressed genes (DEGs) were identified after 24 h of *S. aureus* infection. Two hundred fifty-three genes were significantly changed after 48 h of *S. aureus* infection. STAT1 was consistently up-regulated in these three treatments. *TP53*, *JAK2*, *CEBPA*, *STAT3*, *MYC*, *CTNNB1* and *PRKCA* were only identified in the 8 h or 24 h *S. aureus* infection groups. *CTNNB1* and *PRKCA* were for the first time identified as potential essential genes in *S. aureus* infection of macrophages. In the Gene Ontology (GO) term analysis, the defense response was shown to be the most significantly changed biological process among all processes; KEGG pathway analysis identified the JAK-STAT signaling pathway involved in early infection.

**Conclusions:**

Our systematic analysis identified unique gene expression profiles and specifically changed biological processes of the macrophage response to different *S. aureus* exposure times.

**Electronic supplementary material:**

The online version of this article (10.1186/s11658-018-0090-4) contains supplementary material, which is available to authorized users.

## Background

*Staphylococcus aureus* is one of the leading causes of bacterial infections in humans with an incidence rate from 20 to 50 cases/100,000 population per year, while the attributable mortality is greater than those of AIDS, tuberculosis, and viral hepatitis combined [1]. The Gram-positive bacterium *S. aureus* can express a broad variety of bacterial virulence factors, and recent studies showed that it can survive in several host cells. *S. aureus* can escape from phagocyte monitoring due to its gene mutation [2]. The mutation can also increase its infection ability and antibiotic resistance and no vaccines currently are licensed for *S. aureus* [3]. Long-term exposure to *S. aureus* results in the adaptive immune response and influences the immunological memory establishment and maintenance [4, 5].

Macrophages play a critical role in innate immune responses to bacterial infection. Macrophages are primary professional phagocytes that are designed to devour and kill microbes by pinocytosis, receptor-mediated endocytosis or phagocytosis. Previous studies have identified profound gene expression profile changes in differently activated macrophages [6]. However, the underlying molecular mechanism of the macrophage response to *S. aureus* has still not been completely elucidated.

Recently, Koziel et al. indicated that *S. aureus* might induce cytoprotective mechanisms by regulating the gene expression profiles inside macrophages [7]. However, their study was mainly focused on the mechanism of cell death and apoptosis upon *S. aureus* infection. To further dissect the global gene expression profile change and corresponding signaling pathways in *S. aureus* infection-induced immune response, we systematically analyzed the macrophage gene expression profile to identify genes with significant changes in response to *S. aureus* exposure. We identified several essential genes and pathways during the *S. aureus* infection in our analysis. The results were further confirmed by western blot and qPCR analyses, providing the first molecular targets for macrophage response to *S. aureus* infection.

## Methods

### Data collection, data processing and filtering

To retrieve the human monocyte-derived macrophages (hMDMs) gene expression profile, the Gene Expression Omnibus (GEO) microarray expression dataset (http://www.ncbi.nlm.nih.gov/geo/) was searched and GSE 13670 (http://www.ncbi.nlm.nih.gov/geo/query/acc.cgi?acc=GSE13670) was obtained for our expression analysis. Briefly, control and *S. aureus*-exposed macrophage cells were analyzed at different time points (8, 24 and 48 h after infection) which each includes 5 samples [8]. Unprocessed data (.cel files) were collected. This dataset was Affymetrix Human Genome U133 Plus 2.0. Corresponding probe annotation files were downloaded for further analysis.

The normalization process in GCRMA [9] was used to quantify the microarray signal for our research. The package genefilter [10] in R language was employed to filter out uninformative data, such as control probe sets and other internal controls as well as to remove genes expressed uniformly close to background detection levels. The filter did not remove probe sets without Entrez gene identifiers or with identical Entrez gene identifiers.

### Differentially expressed gene analysis

Three statistical comparisons were carried out as follows. Comparison 1 was conducted between the 8 h infection and control groups (hMDM_SA_8hr vs hMDM_control_8hr), Comparison 2 was made between the 24 h infection and control groups (hMDM_SA_24hr vs hMDM_SA_24hr), and Comparison 3 was made between 48 h infection and control groups (hMDM_SA_48hr vs hMDM_control_48hr). The package limma [11] in R language was used to identify differential expressed genes from these 3 comparisons. For those probes that had an identical Entrez gene identifier, we only kept the probe showing the greatest variance. Genes with |log2(FC)| > 1.5 and the adjusted *p* value < 0.01 were accepted as statistically differentially expressed. The *p* value was adjusted by applying Benjamini and Hochberg’s (BH) false discovery rate (FDR) correction to the original *p* value, and the fold change threshold was set to focus on significantly differentially expressed genes.

The obtained DEGs from three comparisons were compared against each other and the common DEGs in all three comparisons or only in one comparison were identified.

### Hierarchical clustering, GO and KEGG pathway analysis

To better understand the global gene expression patterns, we performed hierarchical clustering [12] to classify analyzed samples based on gene expression profiles. The DEGs were further analyzed using Gene Ontology terms (biological processes) and KEGG pathways. Heatmaps for the DEGs classified in targeted biological processes or KEGG pathways were generated by gplots [13] in the R package.

ClusterProfiler [14] in R packages was used to detect Gene Ontology categories and KEGG pathways with significant overrepresentation in DEGs compared with the whole genome. The significantly enriched biological processes were identified with an adjusted *p* value less than 0.01. For KEGG pathway analysis, the adjusted *p* value was set to less than 0.05.

### Construction of biological network

Protein-protein interaction (PPI) databases were retrieved from HPRD [15], BIOGRID [16], and PIP [17] databases. Pair interactions in any of the three databases were chosen to be included in our curated PPI database. As a result, a total of 561,405 pair interactions were included in our analysis. Cytoscape [18] was used to construct the interaction network. After functional enrichment analysis, the DEGs specified in significantly altered biological processes (Gene Ontology terms) and KEGG pathways were mapped to corresponding networks for interaction analysis. In the network analysis, if the connection of a gene to other genes is more than the average connection, it suggests that this gene has multiple interactions with other genes. Therefore, this gene will be considered as a hub gene compared to other genes.

### siRNA and overexpression experiments

siRNA for Control (D-001810-01-05) and siRNA smart pool targeting beta-catenin (L-040628-00-0005) were purchased from Dharmacon and siRNA transfection using Lipofectamine 2000 (Thermo Fisher Scientific). pcDNA3.1/nV5-DEST-beta catenin (Plasmid #20140) was purchased from Addgene and was also transfected using Lipofectamine 2000.

### Cell culture, western blot and quantitative real-time PCR analysis

RAW 264.7 cells were purchased from the Cell Bank of the Chinese Academy of Sciences and cultured following ATCC conditions. Antibodies for beta-catenin (8480) and beta-actin (3700) were purchased from Cell Signaling.

The TRIzol kit (Invitrogen USA) was used to extract total RNA from cells following the manufacturer’s protocol. cDNA was synthesized using the cDNA synthesis kit (Invitrogen, USA). Quantitative real-time PCR was performed using the TaqMan system (Thermo Fisher Scientific) and 18S rRNA was used as an internal control. All TaqMan probes were purchased from Thermo Fisher Scientific. All setups were performed in triplicate. The results were derived by normalization to 18S rRNA. The *p*-value was set at 0.05.

## Results

### Differential gene expression analysis of macrophages infected by *S. aureus* at various time points

In order to identify essentially and significantly changed genes of the macrophage response to *S. aureus* infection, we analyzed the gene expression profile of the macrophage database which has 3 different *S. aureus* exposure times (GSE 13670). At |log_2_(fold change)| > 1.5 and an adjusted *p* value < 0.01, 624 differentially expressed genes (DEGs) were identified for the first comparison with 8 h of *S. aureus* exposure, among which 418 were up-regulated and 206 were down-regulated. A total of 613 DEGs were identified after 24 h of *S. aureus* infection in Comparison 2 with 370 DEGs up-regulated and 243 DEGs down-regulated. For Comparison 3, a total of 253 DEGs were identified after 48 h of *S. aureus* infection, with 195 DEGs up-regulated and 58 DEGs down-regulated (Table 1).

**Table 1:** Significantly changed genes in *S. aureus-*exposed macrophage

Comparison	DEGs Count |logFC| > 1.5 & adjusted *p* < 0.01	
hMDM_SA_8hr vs hMDM_control_8hr (Comparison 1)	624	418 (up) 206 (down)
hMDM_SA_24hr vs hMDM_SA_24hr (Comparison 2)	613	370 (up) 243(down)
hMDM_SA_48hr vs hMDM_control_48hr (Comparison 3)	253	195 (up) 58 (down)

**Fig. 1:**
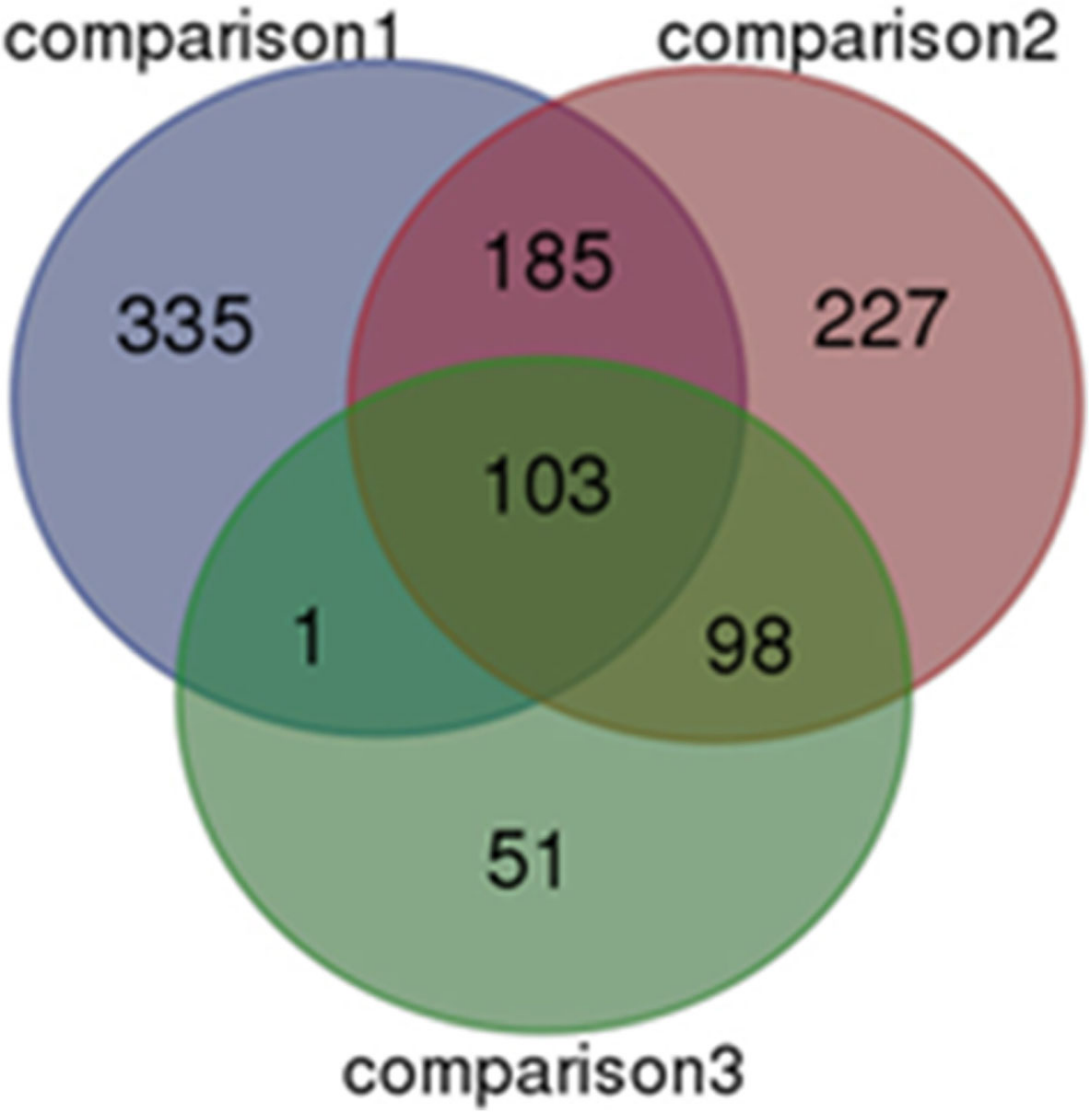
Overlapping DEGs of macrophages from different *S. aureus* exposure times (Comparison 1, 2 and comparison 3)

Overlapping of all the DEGs from three comparisons was performed by Limma [11] to identify the commonly changed genes from different *S. aureus* exposure times (Fig. 1). As shown by the Venn diagram, 103 common genes were significantly changed after 8 h, 24 h or 48 h of *S. aureus* infection. Among these, 94 DEGs were up-regulated and 9 DEGs were down-regulated (Additional file 1: Table S1). There were 335 unique DEGs changed only in Comparison 1, among which 187 DEGs were up-regulated and 148 were down-regulated (Fig. 1, Additional file 2: Table S2). Two hundred twenty-seven DEGs changed only in Comparison 2, among which 76 DEGs were up-regulated and 151 were down-regulated (Fig. 1, Additional file 3: Table S3). Fifty-one DEGs were changed only in Comparison 3, among which 36 DEGs were up-regulated and 15 down-regulated (Fig. 1, Additional file 4: Table S4).

### Construction of biological network

**Fig. 2:**
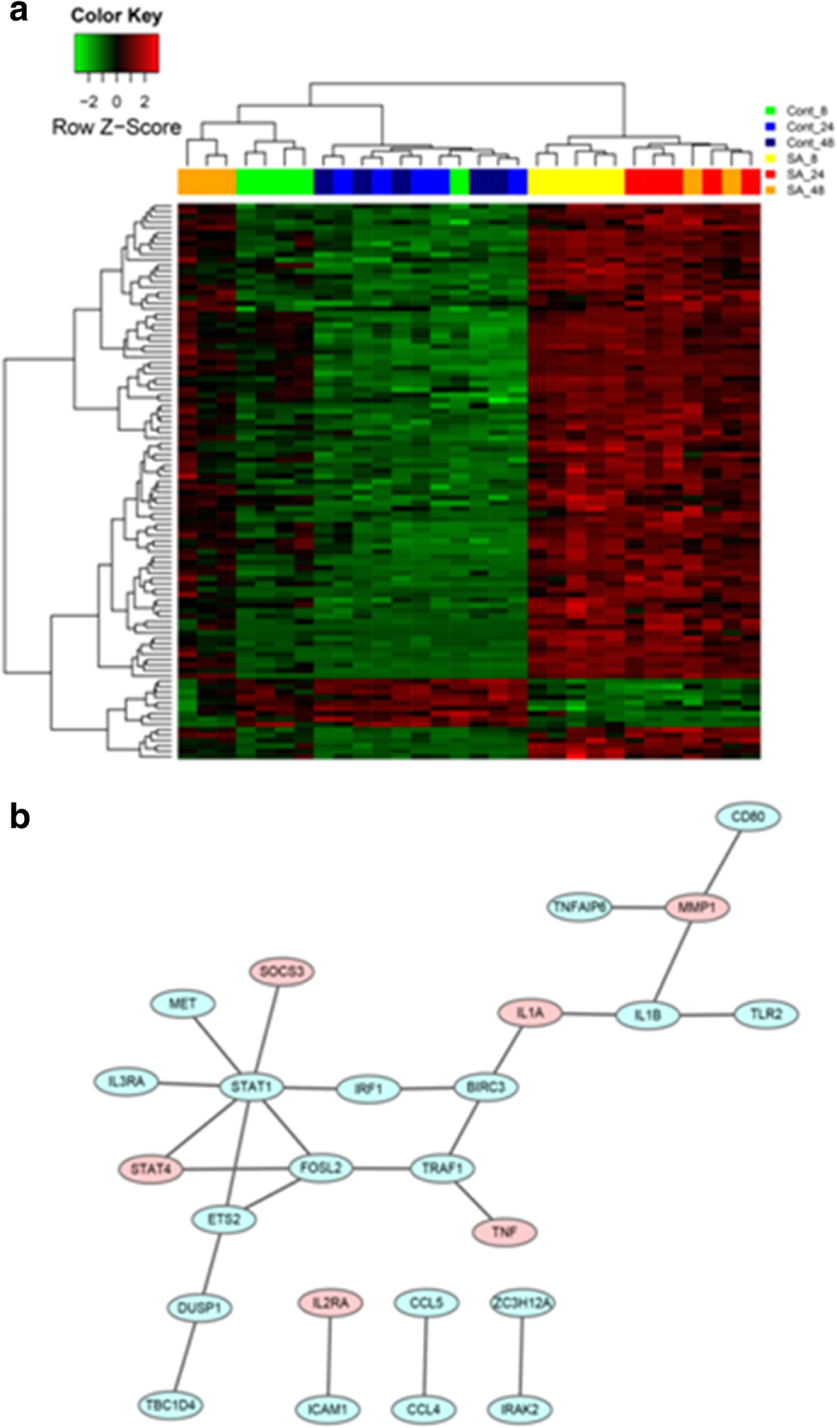
Heat map of common DEGs of all 3 comparisons (**a**) and corresponding biological network (**b**). **a** Heat map of hierarchical clustering in all datasets for common DEGs. “Red”: high expression, “green”: low expression. **b** Biological network constructed according to the direct connection among DEGs. “Red”: up-regulation, and “green”: down-regulation

**Fig. 3:**
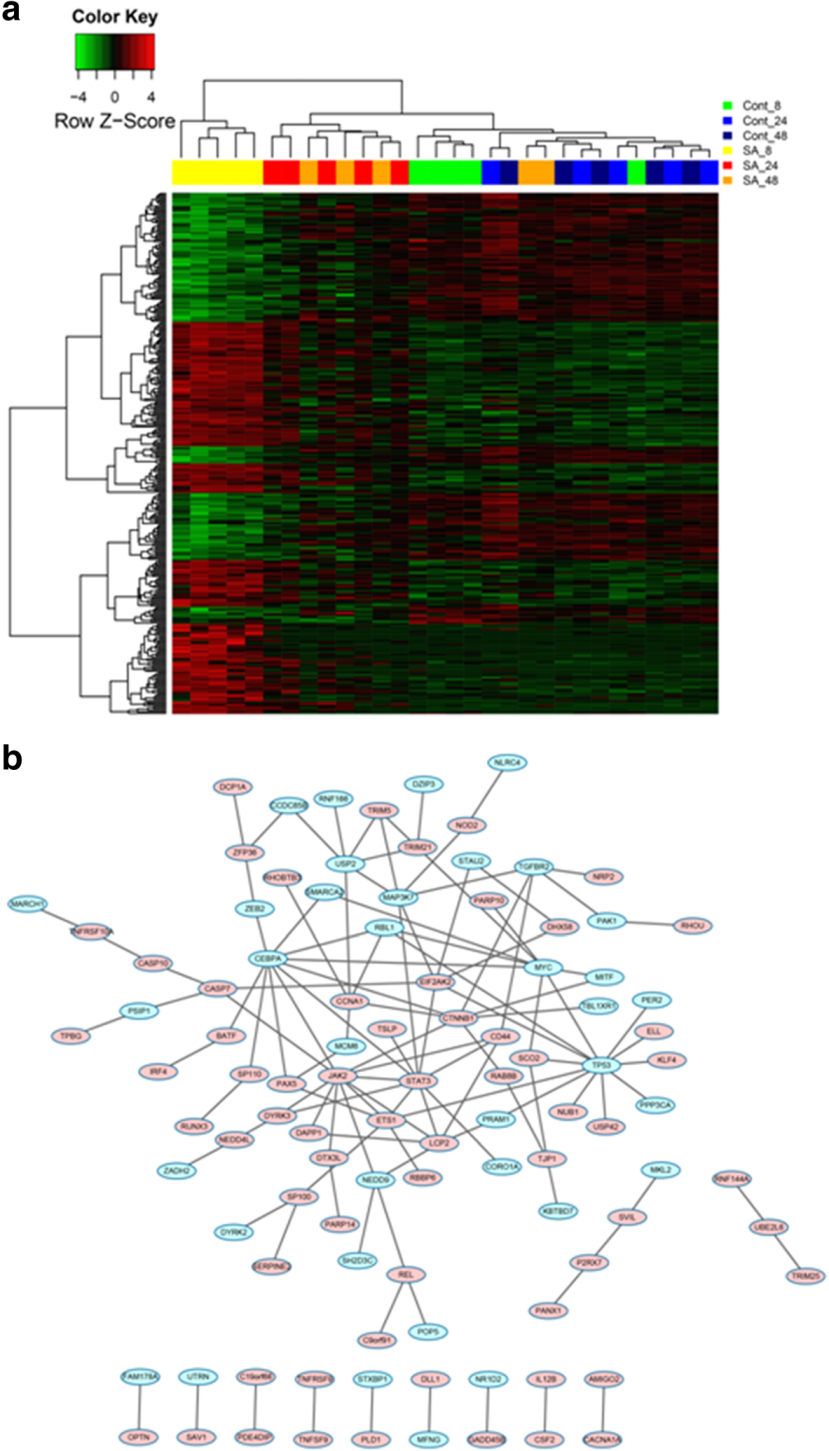
Heat map of common DEGs in Comparison 1 (**a**) and corresponding biological network (**b**). **a** Heat map of hierarchical clustering for common DEGs in Comparison 1. “Red”: high expression, “green”: low expression. **b** Biological network constructed according to the direct connection among DEGs. “Red”: up-regulation, and “green”: down-regulation

**Fig. 4:**
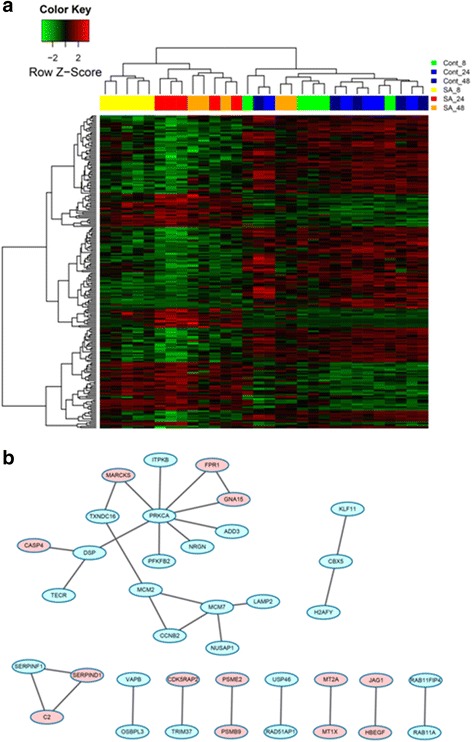
Heat map of common DEGs in Comparison 2 (**a**) and corresponding biological network (**b**). **a** Heat map of hierarchical clustering for common DEGs in Comparison 2. “Red”: high expression, “green”: low expression. **b** Biological network constructed according to the direct connection among DEGs. “Red”: up-regulation, and “green”: down-regulation

**Fig. 5:**
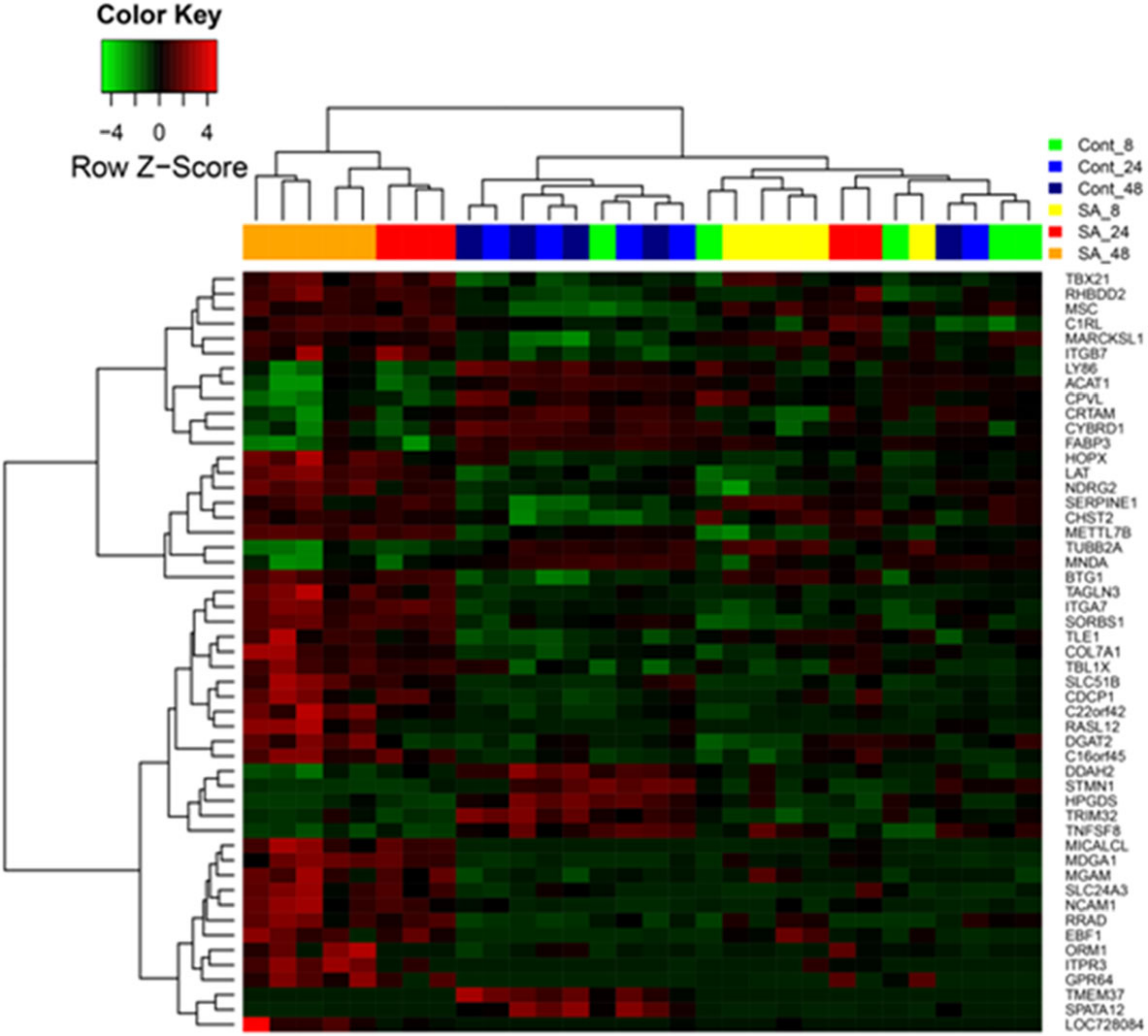
Heat map of hierarchical clustering of common DEGs in Comparison 3. “Red”: high expression, “green”: low expression

Heatmaps or PPI networks were constructed and visualized for these significantly changed genes commonly or specifically changed in these three comparisons (Figs. 2, 3, 4 and 5). Several hub genes which are key members of significant pathways/networks were identified. Among 103 common DEGs, *STAT1* is the hub gene in the constituted network (Fig. 2a and b). *STAT1* is involved in cell growth regulation and antiviral and immune defense, such as inflammation and innate and adaptive immunity, antiproliferative responses and tumor suppression, and it participates in crosstalk with other signal transduction pathways [19]. The expression of *STAT1* was significantly increased among the common genes in our analysis. For DEGs changed only in Comparison 1, the biological network was more complicated and the key genes in the network were *TP53*, *JAK2*, *CEBPA*, *STAT3*, *MYC* and *CTNNB1* (Fig. 3a and b). For DEGs changed only in Comparison 2, the key gene was *PRKCA* (Fig. 4a and b). No biological network was obtained for DEGs changed only in Comparison 3 (Fig. 5).

### GO and KEGG pathway analysis of DEGs in macrophages infected by *S. aureus* with different durations of treatment

To better understand the protein and biological modules involved in the process of macrophages exposed to *S. aureus*, we used ClusterProfiler [14] to perform GO categories and KEGG pathways enrichment analysis with DEGs at different time points. Seven hundred forty-two biological processes and 25 KEGG pathways were enriched in the analysis of these common DEGs. Among them were significantly changed processes or pathways related to infection, such as defense response, immune system process, signal transduction and JAK-STAT signaling pathway (Table 2).

**Table 2:** Significant GO biological processes and KEGG pathways for common DEGs in all three comparisons

	Asjusted *p* value	Count	Term
GO-BP-ID			
GO:0006952	7.13E-17	40	Defense response
GO:0002376	3.88E-15	45	Immune system process
GO:0007165	9.06E-12	61	Signal transduction
KEGG-ID			
04630	2.23E-05	10	Jak-STAT signaling pathway

For the *S. aureus* 8 h treatment group (Comparison 1), 338 biological processes and 9 KEGG pathways were over-represented. Consistent with the results of analysis of common DEGs, these significantly enriched processes and pathways are also related to infection, including defense response, immune system process, myeloid cell differentiation and symbiosis, encompassing mutualism through parasitism, JAK-STAT signaling pathway, apoptosis and cell cycle (Table 3). These pathways and processes might play major roles in the early stage of *S. aureus* infection.

**Table 3:** GO biological processes and KEGG pathways for significantly changed genes in comparison 1

	Adjusted *p* value	Count	Term
GO-BP-ID			
GO:0006952	7.25E-11	60	defense response
GO:0002376	6.03E-09	73	immune system process
GO:0030099	2.51E-08	23	myeloid cell differentiation
GO:0044403	1.52E-04	28	symbiosis, encompassing mutualism through parasitism
KEGG-ID			
04630	1.86E-02	9	Jak-STAT signaling pathway
04210	4.43E-02	6	apoptosis
04110	4.92E-02	7	cell cycle

For 24 h treatment of the *S. aureus* group (Comparison 2), 62 biological processes and 7 KEGG pathways were over-represented. The included significant GO biological processes were “response to stress”, “response to stimulus” and “cell activation” (Table 4). No significant KEGG pathway was identified for Comparison 2. No GO biological processes or KEGG pathways were significantly enriched after the analysis of the DEGs in Comparison 3.

**Table 4:** GO biological processes for significantly changed genes in comparison 2

	Adjusted *p* value	Count	Term
GO-BP-ID			
GO:0006950	1.45E-06	69	response to stress
GO:0050896	1.81E-05	116	response to stimulus
GO:0001775	1.49E-03	23	cell activation

### Cell culture, quantitative real-time PCR and western blot analyses

**Fig. 6:**
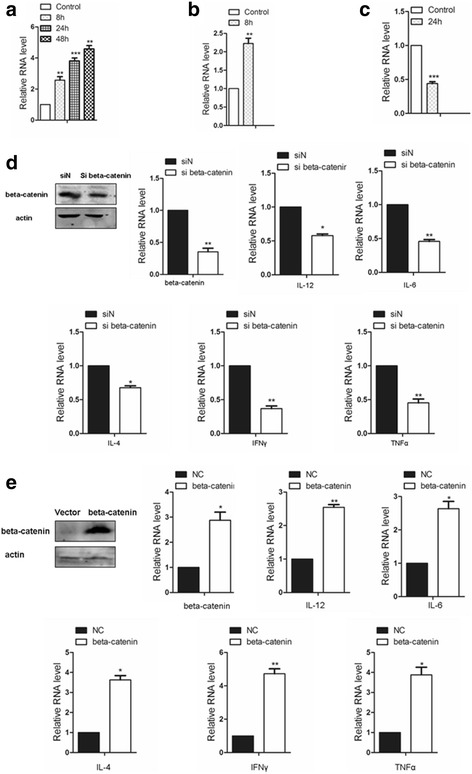
In macrophages with different *S. aureus* exposure times (**a**) STAT1 was significantly increased (8, 24 and 48 h), **b** beta-catenin (CTNNB1) was increased in Comparison 1 (8 h), **c** PRKCA was down-regulated in Comparison 2 (24 h). **d** 48 h after CTNNB1 knockdown, the expression levels for IL-12, IL-6, IL-4, IFNγ, and TNFα were reduced in macrophages by qPCR analysis. **e** IL-12, IL-6, IL-4, IFNγ, and TNFα expression levels were increased after CTNNB1 over-expression in macrophages

To confirm the results obtained from bioinformatic analysis, quantitative real-time PCR was employed to detect the mRNA expression of key genes after different *S. aureus* exposure times in RAW 264.7 murine macrophages. As shown in Fig. 6a, the expression level of the HUB gene STAT1 was significantly increased (2.5, 3.8, and 4.5 fold respectively) at all exposure time settings compared to the control group. While a 2.2-fold increase was observed for beta-catenin (CTNNB1) after 8 h of *S. aureus* exposure, PRKCA was down-regulated (~ 45% compared to control) at the 24 h time point. These results were consistent with previous analyses.

Considering the early change of beta-catenin and the enriched inflammation signaling in murine macrophages after *S. aureus* exposure and the effect of beta-catenin on inflammation signaling [20], we are curious whether beta-catenin was involved in the inflammatory response with cytokine production regulation in a general condition (without *S. aureus* exposure). Here, several typical cytokines, including IL-12, IL-6, IL-4, IFNγ, and TNFα, were examined by qPCR analysis after knockdown of beta-catenin in RAW 264.7 murine macrophages in the general situation (without *S. aureus* exposure). Figure 6d indicates that the knockdown of beta-catenin by siRNA leads to a reduced expression level for IL-12 (55% compared to control), IL-6 (45% compared to control), IL-4 (65% compared to control), IFNγ (40% compared to control), and TNFα (45% compared to control). In contrast, over-expression of CTNNB1 in macrophages leads to the increased expression of IL-12, IL-6, IL-4, IFNγ, and TNFα (Fig. 6e).

## Discussion

*S. aureus* is one of the most frequently isolated pathogens, with significant morbidity and mortality. The modulation of macrophages’ functions by *S. aureus* has a significant impact on the immune response to bacterium infection [7]. Therefore, understanding the molecular mechanism of *S. aureus* infection leading to the cell immune response and apoptosis is of great clinical significance.

In the current study, we analyzed the gene expression profiles of macrophages exposed to *S. aureus* for different durations. Interestingly, we identified more DEGs during the early stage of *S. aureus* infection (624 DEGs and 613 DEGs in Compassion 1 and Comparison 2, respectively) compared to the later stage (253 DEGs in Comparison 3), suggesting that the macrophages underwent more significant gene expression profile changes within 24 h after the *S. aureus* exposure.

We identified eight potential essential genes in all three comparisons or specific in only one comparison. Among them, the hub gene STAT1 was the only one consistently up-regulated from all 3 comparisons, indicating that its expression is constitutively elevated during different *S. aureus* infection times. As far as we know, this is the first molecular evidence that STAT1 is elevated in *S. aureus* infected human monocyte-derived macrophages. The increased STAT1 expression after *S. aureus* exposure was further confirmed by qPCR analysis in murine macrophages (RAW 264.7) at different time points. Similarly, a previous study indicated that STAT1 was involved in *Cryptococcus neoformans* infection in BALB/c mice [21]. The STAT1-mediated signal transduction pathway played an important role in elicitation of the classical macrophage phenotype during *C. neoformans* challenge [22]. Whether the expression of STAT1 is directly related to the absolute number of macrophages is not clear. *S. aureus* infection stimulated the expression of STAT1, which suggested that the STAT1 signaling pathway was activated to rapidly and tightly regulate the macrophage response to the infectious challenges.

The mRNA expression of six key genes was significantly changed after 8 h of *S. aureus* infection. Among them the expression of JAK2, STAT3 and CTNNB1 was up-regulated, while TP53, CEBPA and MYC were consistently down-regulated. As a key component of the JAK2/STAT3/Snail pathway, JAK2 is associated with macrophages on tumor infiltration involved in epithelial-mesenchymal transition [23]. STAT3 mediates the expression of a variety of genes in response to cell stimuli, and plays a key role in many cellular processes, such as cell growth and apoptosis [24]. STAT3 and IL-10 play a key role in driving immune dysregulation and severe immunodeficiency [25]. A recent study suggested that the absence of TP53 in endometrial cells initiates chronic inflammation, and a TP53 mutant in endometrial cancer cells induces normal macrophages to express genes that are involved in the inflammatory reaction through signal pathways [26]. In our study, after 24 h of *S. aureus* infection, the expression of TP53 in macrophages was down-regulated. MYC was down-regulated in our analyses; several studies have already suggested that MYC played a role in apoptosis, the cell cycle and cellular transformation [27, 28]. The expression of CEBPA was also down-regulated during 8 h of *S. aureus* treatment. Its encoded protein can modulate the expression of genes involved in cell cycle regulation [29]. PRKCA is a serine- and threonine-specific protein kinase, which is activated by phosphatidylserine in a calcium-dependent manner. PKC family members phosphorylate a wide variety of protein targets and are known to be involved in diverse cellular signaling pathways. PRKCA was down-regulated after 24 h of *S. aureus* infection. To our knowledge, this is the first time CTNNB1 and PRKCA have been identified as essential genes in the relevant studies. Suppression of PRKCA might play a role in the infection response, which is similarly reported in *Brucella* and some other intracellular pathogens, such as *Salmonella*, *Leishmania*, and *Legionella* infected macrophages [30].

Our KEGG pathways analysis identified the JAK-STAT signaling pathway as the most significant process in the early stage (8 h) of *S. aureus* infection of macrophages. The JAK-STAT pathway is one of several important intracellular signaling pathways responsible for the activation of macrophages, the inflammatory response and inhibition of apoptosis [8, 31]. Similar to our results, Zhu et al. also found that the JAK-STAT signaling pathway was activated during the phagosome maturation of macrophage exposure to *S. aureus* [32].

The Wnt/beta-catenin pathway might also be involved in the macrophage inflammatory response to *S. aureus*. As shown in Fig. 6e, the qPCR analysis in murine macrophages suggested that the elevated expression of CTNNB1 led to upregulation of IL-12, IL-6, IL-4, IFNγ, and TNFα expression levels, further highlighting the importance of CTNNB1 in signaling/activation of the inflammatory response.

## Conclusions

In summary, we comprehensively analyzed the gene expression profiles of *S. aureus* infected macrophages for different durations. Several essential genes were identified in our analysis, such as STAT1, CTNNB1 and PRKCA. As far as we know, CTNNB1 and PRKCA were identified for the first time. The JAK-STAT pathway and Wnt/beta-catenin pathway processes were activated in *S. aureus* infected macrophages. Our findings provide clues to further explore the detail antimicrobial mechanisms of macrophage response to *S. aureus* infection.

## Additional files

Additional file 1: **Table S1.** One hundred three common DEGs from all comparisons (|log2(fold change)| > 1.5 & adjusted *p* value < 0.01). (DOCX 29 kb)

Additional file 2: **Table S2.** Three hundred thirty-five DEGs obtained in Comparison 1 (|log2(fold change)| > 1.5 & adjusted *p* value < 0.01). (DOCX 30 kb)

Additional file 3: **Table S3.** Two hundred twenty-seven DEGs obtained in Comparison 2 (|log2(FC)| > 1.5 & adjusted *p* < 0.01). (DOCX 25 kb)

Additional file 4: **Table S4.** Fifty-one DEGs obtained in Comparison 3 (|log2(fold change)| > 1.5 & adjusted *p* value < 0.01). (DOCX 17 kb)

## Abbreviations

BH: Benjamini and Hochberg’s
DEGs: Differentially expressed genes
FDR: False discovery rate
GEO: Gene Expression Omnibus
GO: Gene Ontology
HPRD: Human protein reference database
KEGG: Kyoto Encyclopedia of Genes and Genomes
PIP: Human protein-protein interaction prediction database
PPI: Protein-protein interaction

## References

[1] Tong SY, Davis JS, Eichenberger E, Holland TL, Fowler VG (2015). *Staphylococcus aureus* infections: epidemiology, pathophysiology, clinical manifestations, and management. Clin Microbiol Rev.

[2] Beth L, Noboru M, Virgin HW (2011). Autophagy in immunity and inflammation. Nature.

[3] Maria Belén M, María Isabel C (2012). *Staphylococcus aureus* promotes autophagy by decreasing intracellular cAMP levels. Autophagy.

[4] von Eiff C, Peters G, Becker K (2006). The small colony variant (SCV) concept—the role of staphylococcal SCVs in persistent infections. Injury.

[5] Wertheim HF (2004). Risk and outcome of nosocomial *Staphylococcus aureus* bacteraemia in nasal carriers versus non-carriers. Lancet.

[6] Gordon S (2003). Alternative activation of macrophages. Nat Rev Immunol.

[7] Koziel J (2009). Phagocytosis of *Staphylococcus aureus* by macrophages exerts cytoprotective effects manifested by the upregulation of antiapoptotic factors. PLoS One.

[8] Gebru E (2011). The role of Janus kinase 2 (JAK2) activation in pneumococcal EstA protein-induced inflammatory response in RAW 264.7 macrophages. Microb Pathog.

[9] Wu J, Irizarry R, Macdonald J, Gentry J. Background adjustment using sequence information. R Package Version. 2005:2.

[10] Hahne F, Huber W, Gentleman R, Falcon S (2010). Bioconductor case studies.

[11] De Groot P, Reiff C, Mayer C, Müller M (2008). NuGO contributions to GenePattern. Genes Nutr.

[12] Tavazoie S, Hughes JD, Campbell MJ, Cho RJ, Church GM (1999). Systematic determination of genetic network architecture. Nat Genet.

[13] Warnes GR, et al. gplots: various R programming tools for plotting data. R Package Version. 2009:2.

[14] Yu G, Wang L-G, Han Y, He Q-Y (2012). clusterProfiler: an R package for comparing biological themes among gene clusters. OMICS.

[15] Prasad TK (2009). Human protein reference database—2009 update. Nucleic Acids Res.

[16] Chatr-Aryamontri A (2013). The BioGRID interaction database: 2013 update. Nucleic Acids Res.

[17] McDowall MD, Scott MS, Barton GJ (2009). PIPs: human protein–protein interaction prediction database. Nucleic Acids Res.

[18] Smoot ME, Ono K, Ruscheinski J, Wang P-L, Ideker T (2011). Cytoscape 2.8: new features for data integration and network visualization. Bioinformatics.

[19] Ramana CV, Chatterjee-Kishore M, Nguyen H, Stark GR (2000). Complex roles of Stat1 in regulating gene expression. Oncogene.

[20] Suryawanshi A, Tadagavadi RK, Swafford D, Manicassamy S (2016). Modulation of inflammatory responses by Wnt/beta-catenin signaling in dendritic cells: a novel immunotherapy target for autoimmunity and cancer. Front Immunol.

[21] Sánchez-Espiridión B (2012). Immunohistochemical markers for tumor associated macrophages and survival in advanced classical Hodgkin’s lymphoma. Haematologica.

[22] Leopold Wager CM (2015). STAT1 signaling within macrophages is required for antifungal activity against *Cryptococcus neoformans*. Infect Immun.

[23] Fu X-T (2015). Macrophage-secreted IL-8 induces epithelial-mesenchymal transition in hepatocellular carcinoma cells by activating the JAK2/STAT3/Snail pathway. Int J Oncol.

[24] Hirano T, Ishihara K, Hibi M (2000). Roles of STAT3 in mediating the cell growth, differentiation and survival signals relayed through the IL-6 family of cytokine receptors. Oncogene.

[25] Krejsgaard T, et al. Staphylococcal enterotoxins promote lymphoma-associated immune dysregulation by modulating benign and malignant T-cell interactions. Blood. 2014; 10.1182/blood-2014-2001-551184.

[26] Stodden G (2015). Loss of Cdh1 and Trp53 in the uterus induces chronic inflammation with modification of tumor microenvironment. Oncogene.

[27] Hsu TY-T (2015). The spliceosome is a therapeutic vulnerability in MYC-driven cancer. Nature.

[28] Quintanilla-Martinez LIX (2015). Is it only about MYC? How to approach the diagnosis of diffuse large B-cell lymphomas. Hematol Oncol.

[29] Kagita S, Uppalapati S, Gundeti S, Digumarti R. Correlation of C/EBPα expression with response and resistance to imatinib in chronic myeloid leukaemia. Jpn J Clin Oncol. 2015; 10.1093/jjco/hyv064. 10.1093/jjco/hyv064 25920395

[30] Cannella AP (2012). Antigen-specific acquired immunity in human brucellosis: implications for diagnosis, prognosis, and vaccine development. Front Cell Infect Microbiol.

[31] Harpur A, Andres A, Ziemiecki A, Aston R, Wilks A (1992). JAK2, a third member of the JAK family of protein tyrosine kinases. Oncogene.

[32] Zhu F, Zhou Y, Jiang C, Zhang X (2015). Role of JAK-STAT signaling in maturation of phagosomes containing *Staphylococcus aureus*. Sci Rep.

