# Supplementary material for: Essential genes of the macrophage response to Staphylococcus aureus exposure
Source: Cell Mol Biol Lett. 2018 May 23;23:25. doi: 10.1186/s11658-018-0090-4 (PMC5966896; doi:10.1186/s11658-018-0090-4)
Supplement: Supplementary file 1 — Table S1. One hundred three common DEGs from all comparisons (|log2(fold change)| > 1.5 & adjusted p value < 0.01). (DOCX 29 kb) [file 11658_2018_90_MOESM1_ESM.docx]

**Table S1. 103 common DEGs from all comparisons (|log2(fold change)| > 1.5 & adjusted p value < 0.01)**

| Gene_symbol | LogFC | P value | Adjusted p value | LogFC | P value | Adjusted p value | LogFC | P value | Adjusted p value |
| --- | --- | --- | --- | --- | --- | --- | --- | --- | --- |
| RSAD2 | 9.08 | 7.26E-15 | 8.26E-12 | 8.09 | 1.11E-13 | 6.06E-10 | 6.88 | 2.48E-11 | 1.35E-07 |
| ISG20 | 7.48 | 6.09E-18 | 4.16E-14 | 5.53 | 9.33E-15 | 1.27E-10 | 2.10 | 5.45E-05 | 2.37E-03 |
| PTGS2 | 7.31 | 2.45E-08 | 1.58E-06 | 7.84 | 1.53E-10 | 9.51E-08 | 5.71 | 7.64E-08 | 3.94E-05 |
| CCR7 | 7.07 | 2.74E-12 | 8.42E-10 | 6.36 | 2.86E-11 | 2.70E-08 | 6.79 | 6.76E-12 | 6.15E-08 |
| LOC440896 | 7.07 | 6.13E-15 | 7.97E-12 | 7.02 | 7.13E-15 | 1.27E-10 | 7.52 | 1.38E-15 | 3.77E-11 |
| IL23A | 6.58 | 1.11E-08 | 8.38E-07 | 4.91 | 1.85E-06 | 7.17E-05 | 3.99 | 3.69E-05 | 1.89E-03 |
| TFPI2 | 6.37 | 2.48E-10 | 3.90E-08 | 6.87 | 5.09E-11 | 4.26E-08 | 5.51 | 4.72E-09 | 5.37E-06 |
| IDO1 | 6.35 | 9.16E-09 | 7.23E-07 | 10.21 | 3.83E-13 | 1.16E-09 | 9.31 | 3.07E-12 | 4.20E-08 |
| SLAMF1 | 6.28 | 2.36E-09 | 2.52E-07 | 5.49 | 3.10E-08 | 3.99E-06 | 3.15 | 1.44E-04 | 4.55E-03 |
| CCL20 | 5.33 | 1.77E-07 | 7.82E-06 | 8.41 | 2.16E-11 | 2.36E-08 | 8.14 | 4.30E-11 | 1.96E-07 |
| IL1A | 5.30 | 1.75E-07 | 7.75E-06 | 6.48 | 4.23E-09 | 9.62E-07 | 3.83 | 2.70E-05 | 1.53E-03 |
| INHBA | 5.30 | 4.94E-08 | 2.82E-06 | 5.97 | 5.19E-09 | 1.13E-06 | 5.14 | 1.69E-06 | 2.56E-04 |
| MMP1 | 5.09 | 1.36E-04 | 1.43E-03 | 6.21 | 1.00E-05 | 2.60E-04 | 4.97 | 1.79E-04 | 5.14E-03 |
| CMPK2 | 5.03 | 6.38E-11 | 1.18E-08 | 4.69 | 2.72E-10 | 1.46E-07 | 4.20 | 2.63E-09 | 3.42E-06 |
| STAT4 | 4.95 | 2.85E-07 | 1.17E-05 | 5.21 | 1.15E-07 | 9.87E-06 | 3.08 | 2.41E-04 | 6.34E-03 |
| LOC731424 | 4.94 | 2.03E-08 | 1.35E-06 | 5.93 | 5.68E-10 | 2.59E-07 | 3.86 | 1.48E-06 | 2.42E-04 |
| LAMP3 | 4.92 | 1.03E-05 | 1.94E-04 | 5.54 | 1.72E-06 | 6.79E-05 | 5.34 | 3.03E-06 | 3.50E-04 |
| IFI27 | 4.79 | 9.91E-08 | 4.97E-06 | 7.04 | 5.01E-11 | 4.26E-08 | 3.63 | 8.95E-06 | 7.12E-04 |
| ITGB8 | 4.68 | 8.73E-08 | 4.51E-06 | 5.51 | 1.53E-11 | 1.99E-08 | 5.10 | 7.88E-11 | 2.64E-07 |
| PDE4B | 4.56 | 2.94E-07 | 1.20E-05 | 5.01 | 5.46E-08 | 5.94E-06 | 4.27 | 8.78E-07 | 1.97E-04 |
| RHOH | 4.53 | 2.04E-07 | 8.75E-06 | 5.31 | 1.14E-08 | 1.84E-06 | 3.06 | 7.22E-05 | 2.85E-03 |
| IL15RA | 4.42 | 8.23E-11 | 1.49E-08 | 4.75 | 1.69E-11 | 2.00E-08 | 3.17 | 5.76E-08 | 3.44E-05 |
| OSM | 4.40 | 3.94E-08 | 2.34E-06 | 5.18 | 1.75E-09 | 5.20E-07 | 3.42 | 2.80E-06 | 3.33E-04 |
| SIGLEC1 | 4.33 | 2.68E-07 | 1.11E-05 | 6.72 | 7.49E-09 | 1.42E-06 | 4.03 | 3.27E-05 | 1.72E-03 |
| TNF | 4.21 | 1.02E-05 | 1.91E-04 | 5.04 | 6.34E-07 | 3.34E-05 | 4.28 | 8.03E-06 | 6.71E-04 |
| SERPINB2 | 4.12 | 1.20E-05 | 2.19E-04 | 7.00 | 1.22E-09 | 4.02E-07 | 6.90 | 1.62E-09 | 2.46E-06 |
| WNT5A | 4.09 | 1.22E-07 | 5.84E-06 | 5.90 | 5.32E-09 | 1.14E-06 | 5.05 | 9.81E-08 | 4.78E-05 |
| IL2RA | 4.08 | 2.88E-06 | 7.25E-05 | 5.86 | 4.94E-09 | 1.10E-06 | 3.95 | 4.62E-06 | 4.59E-04 |
| IL7R | 4.05 | 2.10E-06 | 5.75E-05 | 5.24 | 2.55E-08 | 3.46E-06 | 5.21 | 1.40E-07 | 5.68E-05 |
| TNIP3 | 4.02 | 1.25E-05 | 2.26E-04 | 4.77 | 9.10E-07 | 4.32E-05 | 3.48 | 8.34E-05 | 3.13E-03 |
| FAM124A | 4.01 | 2.14E-09 | 2.35E-07 | 4.99 | 2.24E-11 | 2.36E-08 | 3.39 | 5.14E-08 | 3.19E-05 |
| SOCS3 | 4.00 | 1.59E-10 | 2.70E-08 | 4.91 | 8.89E-10 | 3.43E-07 | 3.18 | 2.24E-06 | 2.89E-04 |
| CXCL2 | 3.99 | 4.12E-04 | 3.35E-03 | 7.32 | 6.91E-08 | 7.13E-06 | 5.79 | 3.39E-06 | 3.75E-04 |
| VEGFA | 3.93 | 2.64E-07 | 1.10E-05 | 3.70 | 7.46E-07 | 3.71E-05 | 2.76 | 5.35E-05 | 2.34E-03 |
| GPR132 | 3.81 | 7.82E-12 | 2.02E-09 | 3.05 | 8.91E-10 | 3.43E-07 | 1.66 | 2.81E-05 | 1.57E-03 |
| NAMPT | 3.78 | 3.71E-08 | 2.22E-06 | 4.34 | 2.60E-09 | 6.57E-07 | 3.13 | 9.09E-07 | 1.99E-04 |
| CXCL3 | 3.74 | 3.97E-04 | 3.27E-03 | 7.56 | 1.02E-08 | 1.70E-06 | 6.62 | 1.16E-07 | 5.29E-05 |
| CCL1 | 3.73 | 2.83E-04 | 2.51E-03 | 7.08 | 1.91E-08 | 2.72E-06 | 6.02 | 3.45E-07 | 1.04E-04 |
| CD38 | 3.72 | 2.71E-04 | 2.43E-03 | 5.90 | 4.37E-07 | 2.49E-05 | 4.83 | 9.91E-06 | 7.64E-04 |
| EREG | 3.65 | 8.92E-05 | 1.03E-03 | 6.02 | 4.01E-08 | 4.85E-06 | 4.75 | 2.24E-06 | 2.89E-04 |
| CKB | 3.63 | 1.13E-11 | 2.72E-09 | 2.77 | 3.29E-09 | 7.91E-07 | 2.11 | 4.62E-07 | 1.30E-04 |
| SGPP2 | 3.52 | 2.04E-05 | 3.26E-04 | 4.42 | 6.50E-07 | 3.40E-05 | 3.90 | 8.34E-06 | 6.85E-04 |
| CSRNP1 | 3.50 | 3.02E-11 | 6.30E-09 | 3.00 | 7.54E-10 | 3.19E-07 | 1.80 | 6.68E-06 | 6.01E-04 |
| ABTB2 | 3.47 | 1.96E-10 | 3.17E-08 | 2.29 | 5.03E-07 | 2.73E-05 | 1.54 | 1.47E-04 | 4.60E-03 |
| SLC1A2 | 3.37 | 7.25E-06 | 1.47E-04 | 2.81 | 8.46E-05 | 1.27E-03 | 3.10 | 2.43E-05 | 1.42E-03 |
| MYO1G | 3.36 | 1.50E-05 | 2.59E-04 | 5.93 | 2.03E-10 | 1.16E-07 | 4.05 | 2.93E-07 | 9.77E-05 |
| NT5E | 3.34 | 4.02E-06 | 9.44E-05 | 6.52 | 5.23E-09 | 1.13E-06 | 5.58 | 9.57E-08 | 4.75E-05 |
| GBP1 | 3.33 | 1.30E-08 | 9.36E-07 | 3.69 | 7.68E-10 | 3.19E-07 | 2.01 | 1.17E-05 | 8.76E-04 |
| CFB | 3.32 | 6.11E-07 | 2.14E-05 | 4.60 | 1.54E-09 | 4.84E-07 | 3.76 | 7.14E-08 | 3.94E-05 |
| IL1B | 3.32 | 2.97E-05 | 4.40E-04 | 7.38 | 5.07E-11 | 4.26E-08 | 6.79 | 2.96E-10 | 7.36E-07 |
| TM4SF1 | 3.30 | 7.29E-05 | 8.87E-04 | 3.96 | 2.60E-06 | 9.28E-05 | 2.94 | 1.48E-04 | 4.60E-03 |
| CXCL8 | 3.17 | 4.01E-05 | 5.53E-04 | 5.74 | 1.94E-09 | 5.51E-07 | 5.14 | 1.68E-08 | 1.39E-05 |
| GCH1 | 3.12 | 7.17E-07 | 2.42E-05 | 4.60 | 5.09E-10 | 2.36E-07 | 3.48 | 1.08E-07 | 4.99E-05 |
| CCL5 | 3.06 | 1.20E-05 | 2.19E-04 | 3.99 | 4.07E-08 | 4.87E-06 | 4.11 | 2.36E-08 | 1.73E-05 |
| IRF1 | 3.04 | 2.98E-07 | 1.21E-05 | 2.94 | 5.29E-07 | 2.85E-05 | 1.79 | 4.43E-04 | 9.91E-03 |
| TNFAIP6 | 3.04 | 5.07E-04 | 3.94E-03 | 5.08 | 4.64E-07 | 2.59E-05 | 4.93 | 7.65E-07 | 1.82E-04 |
| ETS2 | 2.95 | 6.02E-08 | 3.33E-06 | 3.43 | 3.33E-09 | 7.91E-07 | 2.86 | 1.03E-07 | 4.91E-05 |
| PTGES | 2.94 | 2.32E-05 | 3.61E-04 | 5.31 | 9.34E-10 | 3.49E-07 | 5.42 | 6.08E-10 | 1.13E-06 |
| CD80 | 2.90 | 2.11E-08 | 1.39E-06 | 2.63 | 1.22E-07 | 1.02E-05 | 3.42 | 9.66E-05 | 3.45E-03 |
| DUSP5 | 2.89 | 1.77E-08 | 1.21E-06 | 2.75 | 4.44E-08 | 5.22E-06 | 2.58 | 1.37E-07 | 5.68E-05 |
| LOC285957 | 2.71 | 8.78E-06 | 1.71E-04 | 5.25 | 5.15E-11 | 4.26E-08 | 2.58 | 1.76E-05 | 1.15E-03 |
| TRAF1 | 2.63 | 1.38E-06 | 4.13E-05 | 2.51 | 3.90E-07 | 2.29E-05 | 2.10 | 6.86E-06 | 6.10E-04 |
| RCAN1 | 2.62 | 5.83E-08 | 3.27E-06 | 2.20 | 1.06E-08 | 1.73E-06 | 1.63 | 1.89E-06 | 2.67E-04 |
| HS3ST3B1 | 2.61 | 1.42E-04 | 1.47E-03 | 4.14 | 1.66E-07 | 1.27E-05 | 3.86 | 2.48E-06 | 3.09E-04 |
| MAMLD1 | 2.58 | 2.19E-07 | 9.25E-06 | 2.81 | 4.74E-08 | 5.39E-06 | 1.64 | 1.61E-04 | 4.84E-03 |
| MCOLN2 | 2.51 | 3.26E-04 | 2.79E-03 | 3.62 | 2.51E-06 | 8.99E-05 | 2.82 | 8.22E-05 | 3.12E-03 |
| IL3RA | 2.50 | 5.17E-04 | 4.00E-03 | 4.51 | 1.31E-07 | 1.08E-05 | 2.94 | 8.16E-05 | 3.10E-03 |
| HEY1 | 2.50 | 2.42E-04 | 2.23E-03 | 2.85 | 4.94E-05 | 8.48E-04 | 2.73 | 1.19E-04 | 4.00E-03 |
| LHFP | 2.48 | 8.80E-08 | 4.53E-06 | 2.19 | 7.21E-07 | 3.64E-05 | 1.57 | 8.52E-05 | 3.17E-03 |
| SIPA1L1 | 2.48 | 7.57E-08 | 4.02E-06 | 2.59 | 3.30E-08 | 4.19E-06 | 1.52 | 1.19E-04 | 4.00E-03 |
| DUSP1 | 2.47 | 6.49E-07 | 2.23E-05 | 2.72 | 1.27E-07 | 1.04E-05 | 1.73 | 1.05E-04 | 3.64E-03 |
| FPR2 | 2.44 | 5.83E-05 | 7.46E-04 | 4.93 | 1.22E-09 | 4.02E-07 | 3.26 | 2.07E-06 | 2.84E-04 |
| CCL4 | 2.41 | 1.30E-03 | 8.20E-03 | 4.17 | 1.34E-06 | 5.65E-05 | 3.64 | 1.06E-05 | 8.01E-04 |
| G0S2 | 2.40 | 6.70E-04 | 4.90E-03 | 2.83 | 1.13E-04 | 1.57E-03 | 2.70 | 1.89E-04 | 5.37E-03 |
| FOSL2 | 2.36 | 2.08E-07 | 8.89E-06 | 2.53 | 5.98E-08 | 6.35E-06 | 1.63 | 9.48E-05 | 3.41E-03 |
| NAV2 | 2.34 | 5.62E-07 | 2.00E-05 | 2.46 | 2.54E-07 | 1.75E-05 | 2.82 | 2.19E-08 | 1.71E-05 |
| ZC3H12A | 2.13 | 1.11E-06 | 3.47E-05 | 2.40 | 1.51E-07 | 1.19E-05 | 1.63 | 5.20E-05 | 2.30E-03 |
| LAMB3 | 2.09 | 1.54E-03 | 9.34E-03 | 3.84 | 6.76E-07 | 3.50E-05 | 3.06 | 2.09E-05 | 1.29E-03 |
| ITGB3 | 2.00 | 9.93E-04 | 6.63E-03 | 3.04 | 3.11E-07 | 1.98E-05 | 2.06 | 1.71E-06 | 2.56E-04 |
| LY6E | 1.96 | 3.42E-05 | 4.92E-04 | 2.87 | 9.24E-08 | 8.70E-06 | 1.63 | 3.14E-04 | 7.70E-03 |
| KDM6B | 1.93 | 1.22E-05 | 2.21E-04 | 2.06 | 4.97E-06 | 1.54E-04 | 1.59 | 1.47E-04 | 4.59E-03 |
| STAT1 | 1.92 | 1.03E-06 | 3.29E-05 | 2.96 | 3.36E-10 | 1.70E-07 | 1.54 | 2.89E-05 | 1.59E-03 |
| CLCF1 | 1.90 | 1.55E-04 | 1.59E-03 | 2.31 | 1.22E-05 | 3.00E-04 | 2.60 | 2.09E-06 | 2.84E-04 |
| IER3 | 1.87 | 1.13E-03 | 7.33E-03 | 2.60 | 2.71E-05 | 5.50E-04 | 2.34 | 1.05E-04 | 3.64E-03 |
| IRAK2 | 1.85 | 1.09E-05 | 2.02E-04 | 2.53 | 1.16E-05 | 2.89E-04 | 1.95 | 3.05E-04 | 7.56E-03 |
| TLR2 | 1.83 | 3.75E-05 | 5.27E-04 | 3.07 | 8.73E-09 | 1.59E-06 | 2.16 | 3.83E-06 | 4.05E-04 |
| SCARF1 | 1.78 | 6.06E-06 | 1.29E-04 | 3.09 | 1.01E-07 | 9.03E-06 | 2.08 | 4.40E-05 | 2.06E-03 |
| BIRC3 | 1.68 | 2.27E-06 | 6.10E-05 | 2.18 | 4.91E-07 | 2.70E-05 | 2.20 | 4.19E-07 | 1.20E-04 |
| MET | 1.60 | 9.08E-05 | 1.05E-03 | 2.54 | 3.09E-04 | 3.36E-03 | 2.82 | 8.93E-05 | 3.28E-03 |
| C1QTNF1 | 1.57 | 1.44E-03 | 8.89E-03 | 3.10 | 1.69E-07 | 1.28E-05 | 2.17 | 4.02E-05 | 1.97E-03 |
| OGFRL1 | 1.57 | 1.35E-08 | 9.72E-07 | 2.27 | 6.37E-12 | 1.02E-08 | 1.57 | 1.25E-08 | 1.14E-05 |
| ICAM1 | 1.56 | 8.85E-07 | 2.87E-05 | 2.27 | 8.51E-10 | 3.43E-07 | 1.55 | 9.17E-07 | 1.99E-04 |
| SH3PXD2B | 1.54 | 1.01E-05 | 1.91E-04 | 2.22 | 2.26E-08 | 3.15E-06 | 2.10 | 6.29E-08 | 3.66E-05 |
| ARMC9 | 1.51 | 2.61E-04 | 2.36E-03 | 2.66 | 2.51E-09 | 6.48E-07 | 2.10 | 4.36E-09 | 5.17E-06 |
| TBC1D4 | -1.56 | 6.10E-04 | 4.56E-03 | -2.87 | 1.16E-07 | 9.87E-06 | -2.19 | 9.10E-06 | 7.20E-04 |
| RGS18 | -1.69 | 3.19E-04 | 2.75E-03 | -3.14 | 3.52E-08 | 4.37E-06 | -3.01 | 7.55E-08 | 3.94E-05 |
| CDCA7L | -1.94 | 1.26E-03 | 7.99E-03 | -2.62 | 4.44E-05 | 7.85E-04 | -2.43 | 1.15E-04 | 3.92E-03 |
| CTDSPL | -1.94 | 2.91E-05 | 4.32E-04 | -2.63 | 2.73E-07 | 1.82E-05 | -2.25 | 9.45E-07 | 2.01E-04 |
| PRKACB | -1.98 | 1.31E-03 | 8.24E-03 | -2.91 | 1.55E-05 | 3.58E-04 | -2.62 | 7.35E-05 | 2.89E-03 |
| PGBD5 | -2.03 | 3.70E-04 | 3.09E-03 | -3.33 | 3.90E-07 | 2.29E-05 | -2.70 | 1.05E-05 | 7.97E-04 |
| ZNF652 | -2.11 | 1.32E-07 | 6.26E-06 | -1.66 | 2.61E-06 | 9.30E-05 | -1.51 | 2.51E-05 | 1.45E-03 |
| GAS2L3 | -2.45 | 1.23E-06 | 3.76E-05 | -2.41 | 1.56E-06 | 6.31E-05 | -3.12 | 2.00E-06 | 2.77E-04 |
| GJB6 | -2.68 | 7.90E-04 | 5.57E-03 | -3.84 | 1.03E-05 | 2.66E-04 | -3.19 | 1.22E-04 | 4.03E-03 |
