# Supplementary material for: Essential genes of the macrophage response to Staphylococcus aureus exposure
Source: Cell Mol Biol Lett. 2018 May 23;23:25. doi: 10.1186/s11658-018-0090-4 (PMC5966896; doi:10.1186/s11658-018-0090-4)
Supplement: Supplementary file 2 — Table S2. Three hundred thirty-five DEGs obtained in Comparison 1 (|log2(fold change)| > 1.5 & adjusted p value < 0.01). (DOCX 30 kb) [file 11658_2018_90_MOESM2_ESM.docx]

**Table S2. 335 DEGs obtained in comparison 1 (|log2(fold change)| > 1.5 & adjusted p value < 0.01)**

| Gene_symbol | LogFC | P value | Adjusted p value |
| --- | --- | --- | --- |
| ABCC5 | -1.95 | 7.64E-06 | 1.53E-04 |
| ABHD10 | -2.14 | 6.21E-07 | 2.15E-05 |
| ABHD6 | -1.61 | 2.52E-05 | 3.85E-04 |
| ADA | 2.65 | 1.66E-03 | 9.90E-03 |
| ADAM28 | -1.57 | 5.97E-04 | 4.49E-03 |
| ADPRHL2 | 1.58 | 3.06E-09 | 3.02E-07 |
| AMIGO2 | 3.13 | 2.92E-09 | 2.91E-07 |
| ANAPC7 | -1.62 | 2.97E-04 | 2.60E-03 |
| ANKRD37 | 1.72 | 2.60E-06 | 6.74E-05 |
| ANP32A | -2.37 | 2.61E-10 | 4.02E-08 |
| ANXA2R | 1.70 | 2.14E-04 | 2.02E-03 |
| APOBEC3G | 1.86 | 1.53E-06 | 4.48E-05 |
| APOL2 | 1.63 | 3.04E-07 | 1.23E-05 |
| APOL4 | 2.35 | 3.16E-04 | 2.73E-03 |
| APPL2 | -1.84 | 4.26E-06 | 9.85E-05 |
| ARHGAP15 | -2.40 | 1.91E-07 | 8.33E-06 |
| ARHGAP18 | -1.78 | 1.63E-08 | 1.13E-06 |
| ARHGAP26 | -1.77 | 1.47E-05 | 2.56E-04 |
| ARHGAP35 | -1.96 | 1.70E-08 | 1.17E-06 |
| ARHGEF40 | -1.52 | 1.71E-04 | 1.71E-03 |
| ARID5A | 2.16 | 4.83E-13 | 2.10E-10 |
| ARL5B | 2.42 | 4.47E-09 | 4.08E-07 |
| ARRB1 | -1.67 | 5.02E-07 | 1.83E-05 |
| ASB13 | -1.94 | 6.22E-08 | 3.41E-06 |
| ASCL2 | 1.81 | 4.95E-04 | 3.87E-03 |
| ASPH | -1.61 | 5.28E-05 | 6.92E-04 |
| ASPHD2 | 2.10 | 1.62E-05 | 2.76E-04 |
| ATF7IP2 | 2.60 | 1.76E-09 | 1.95E-07 |
| ATG16L2 | -2.00 | 5.00E-07 | 1.82E-05 |
| ATG4C | -1.57 | 6.40E-05 | 8.04E-04 |
| B3GNT7 | 3.73 | 2.47E-09 | 2.60E-07 |
| BATF | 1.90 | 8.96E-06 | 1.73E-04 |
| BLZF1 | 1.79 | 5.70E-11 | 1.09E-08 |
| BMPR2 | 2.04 | 2.66E-10 | 4.06E-08 |
| BPGM | 1.89 | 1.95E-11 | 4.41E-09 |
| BRE-AS1 | 2.52 | 8.89E-08 | 4.55E-06 |
| C11orf96 | 6.31 | 3.45E-09 | 3.32E-07 |
| C15orf61 | -1.51 | 4.26E-08 | 2.48E-06 |
| C16orf54 | -2.68 | 3.99E-08 | 2.36E-06 |
| C19orf66 | 2.67 | 2.32E-15 | 3.96E-12 |
| C1orf53 | -1.53 | 8.45E-04 | 5.86E-03 |
| C2CD2 | -1.66 | 1.26E-06 | 3.86E-05 |
| C2orf43 | -1.52 | 1.12E-05 | 2.07E-04 |
| C3orf58 | -1.53 | 2.10E-06 | 5.76E-05 |
| C5orf56 | 2.50 | 3.11E-08 | 1.91E-06 |
| C9orf91 | 2.07 | 8.02E-07 | 2.64E-05 |
| CACNA1A | 1.75 | 1.52E-05 | 2.61E-04 |
| CASD1 | -1.72 | 5.86E-05 | 7.48E-04 |
| CASP10 | 2.61 | 6.53E-10 | 8.30E-08 |
| CASP7 | 1.75 | 1.14E-11 | 2.74E-09 |
| CATSPERB | 1.63 | 2.25E-09 | 2.45E-07 |
| CCDC85B | -1.51 | 9.80E-05 | 1.11E-03 |
| CCNA1 | 3.37 | 1.04E-05 | 1.95E-04 |
| CCSAP | -1.77 | 1.43E-09 | 1.64E-07 |
| CD1D | -2.00 | 1.52E-03 | 9.23E-03 |
| CD300LB | -1.95 | 9.61E-04 | 6.47E-03 |
| CD44 | 2.11 | 4.87E-05 | 6.48E-04 |
| CD69 | 3.37 | 5.04E-05 | 6.66E-04 |
| CD97 | 1.52 | 1.88E-06 | 5.26E-05 |
| CDYL2 | 2.08 | 3.26E-09 | 3.20E-07 |
| CEBPA | -1.78 | 2.85E-09 | 2.88E-07 |
| CHD9 | -2.10 | 3.28E-08 | 2.00E-06 |
| CLEC2D | 1.66 | 1.19E-03 | 7.63E-03 |
| CLEC4A | -2.20 | 1.96E-05 | 3.16E-04 |
| CLMN | -1.65 | 3.41E-04 | 2.89E-03 |
| CMTR1 | 1.93 | 2.09E-12 | 6.97E-10 |
| COL5A3 | 1.83 | 3.95E-04 | 3.26E-03 |
| CORO1A | -1.59 | 5.93E-05 | 7.56E-04 |
| CPEB3 | 1.66 | 1.07E-05 | 1.99E-04 |
| CSF2 | 6.42 | 4.50E-09 | 4.10E-07 |
| CTNNB1 | 1.59 | 8.08E-06 | 1.60E-04 |
| CXCL10 | 8.00 | 5.35E-10 | 7.16E-08 |
| CXCL11 | 5.07 | 3.09E-08 | 1.90E-06 |
| DAPP1 | 1.71 | 1.42E-06 | 4.24E-05 |
| DCP1A | 1.92 | 4.28E-11 | 8.66E-09 |
| DENND5A | 1.77 | 2.32E-04 | 2.15E-03 |
| DENND5B | -1.54 | 2.24E-05 | 3.51E-04 |
| DHRS9 | -1.57 | 2.96E-05 | 4.39E-04 |
| DHX58 | 2.85 | 5.21E-13 | 2.16E-10 |
| DLL1 | 1.62 | 2.98E-04 | 2.61E-03 |
| DNAJC6 | 1.81 | 1.16E-05 | 2.13E-04 |
| DTX3L | 1.93 | 5.20E-13 | 2.16E-10 |
| DUSP7 | -2.40 | 1.99E-08 | 1.33E-06 |
| DYRK2 | -1.92 | 5.94E-10 | 7.83E-08 |
| DYRK3 | 1.51 | 2.30E-08 | 1.49E-06 |
| DZIP3 | -1.59 | 5.99E-04 | 4.50E-03 |
| EIF2AK2 | 2.86 | 1.45E-14 | 1.23E-11 |
| ELL | 2.05 | 5.13E-06 | 1.14E-04 |
| ERV3-2 | 2.08 | 3.21E-05 | 4.67E-04 |
| ETS1 | 1.77 | 1.53E-03 | 9.26E-03 |
| EVI2B | -1.81 | 3.55E-10 | 5.21E-08 |
| FAM13A | -2.71 | 6.67E-05 | 8.29E-04 |
| FAM178A | -1.61 | 1.22E-06 | 3.73E-05 |
| FAM199X | -1.51 | 1.83E-06 | 5.15E-05 |
| FAM214A | -1.75 | 1.35E-07 | 6.34E-06 |
| FAM216A | -1.81 | 2.85E-04 | 2.52E-03 |
| FAM46A | 1.81 | 2.07E-04 | 1.96E-03 |
| FERMT2 | 1.97 | 1.25E-05 | 2.26E-04 |
| FGD2 | 2.56 | 1.73E-08 | 1.18E-06 |
| FILIP1L | -2.10 | 3.53E-05 | 5.04E-04 |
| FLI1 | -1.73 | 1.78E-05 | 2.94E-04 |
| FLJ32255 | 1.99 | 3.41E-07 | 1.35E-05 |
| FRY | -1.69 | 6.07E-04 | 4.55E-03 |
| FUT8 | -1.51 | 1.37E-04 | 1.44E-03 |
| GADD45B | 3.44 | 1.87E-12 | 6.45E-10 |
| GALNT3 | 2.21 | 3.28E-07 | 1.30E-05 |
| GFPT2 | 3.22 | 3.75E-06 | 8.94E-05 |
| GLCE | -2.03 | 4.22E-08 | 2.47E-06 |
| GMPR | 3.04 | 2.02E-04 | 1.93E-03 |
| GNAQ | -1.61 | 2.20E-10 | 3.53E-08 |
| GNB4 | 1.56 | 7.03E-10 | 8.80E-08 |
| GPATCH11 | -1.54 | 1.38E-08 | 9.86E-07 |
| GPC3 | 2.21 | 9.66E-06 | 1.84E-04 |
| GPR157 | 2.20 | 1.41E-07 | 6.53E-06 |
| GTPBP1 | 2.02 | 3.43E-10 | 5.09E-08 |
| GTPBP2 | 1.86 | 6.75E-07 | 2.31E-05 |
| HAPLN3 | 2.95 | 8.00E-08 | 4.18E-06 |
| HCP5 | -1.71 | 1.18E-08 | 8.72E-07 |
| HENMT1 | -1.67 | 4.60E-06 | 1.04E-04 |
| HES4 | 4.76 | 6.10E-18 | 4.16E-14 |
| HMBS | -1.74 | 2.84E-05 | 4.25E-04 |
| HNMT | -2.56 | 7.33E-08 | 3.93E-06 |
| IFT80 | -2.04 | 1.66E-07 | 7.44E-06 |
| IL12B | 6.64 | 3.80E-09 | 3.62E-07 |
| IL22 | 3.32 | 2.40E-06 | 6.37E-05 |
| IL27RA | -2.74 | 3.32E-08 | 2.01E-06 |
| IL36G | 2.22 | 7.81E-06 | 1.55E-04 |
| IL7 | 1.75 | 1.21E-09 | 1.41E-07 |
| INPP4A | -2.37 | 1.95E-08 | 1.31E-06 |
| INPP5F | -1.68 | 1.77E-05 | 2.94E-04 |
| IRF4 | 2.74 | 6.01E-04 | 4.52E-03 |
| ITGB2-AS1 | -1.90 | 2.84E-04 | 2.51E-03 |
| ITPRIPL2 | 1.70 | 1.18E-08 | 8.74E-07 |
| IVD | -1.54 | 2.49E-04 | 2.27E-03 |
| JAK2 | 1.72 | 1.13E-08 | 8.49E-07 |
| KBTBD7 | -1.68 | 1.97E-05 | 3.17E-04 |
| KCNE3 | -2.40 | 5.61E-08 | 3.17E-06 |
| KCNMB1 | 1.55 | 7.68E-07 | 2.56E-05 |
| KIAA0040 | 3.27 | 5.56E-12 | 1.55E-09 |
| KIAA0226 | 2.13 | 7.37E-10 | 9.19E-08 |
| KIAA0513 | -2.73 | 3.10E-07 | 1.25E-05 |
| KLF4 | 2.08 | 2.27E-04 | 2.12E-03 |
| KLHL24 | -1.59 | 1.62E-03 | 9.70E-03 |
| LACC1 | -1.62 | 7.01E-04 | 5.08E-03 |
| LCP2 | 1.97 | 8.44E-13 | 3.34E-10 |
| LDLR | 1.97 | 6.32E-04 | 4.69E-03 |
| LIF | 3.34 | 3.23E-04 | 2.78E-03 |
| LINC00158 | 3.62 | 1.62E-10 | 2.73E-08 |
| LINC00622 | -3.18 | 5.57E-07 | 1.99E-05 |
| LOC100506585 | -2.39 | 4.14E-04 | 3.36E-03 |
| LOC100507403 | 1.70 | 6.37E-06 | 1.33E-04 |
| LPCAT4 | -1.57 | 4.46E-06 | 1.02E-04 |
| LRRC25 | -2.00 | 1.07E-07 | 5.29E-06 |
| LSS | 2.25 | 6.03E-06 | 1.28E-04 |
| LYRM7 | -1.83 | 3.81E-08 | 2.27E-06 |
| MAP3K7 | -1.76 | 5.36E-07 | 1.93E-05 |
| 1-Mar | -1.57 | 1.20E-04 | 1.30E-03 |
| MASTL | 2.23 | 3.89E-12 | 1.15E-09 |
| MB21D1 | 2.74 | 1.53E-11 | 3.51E-09 |
| MB21D2 | 1.63 | 2.08E-05 | 3.31E-04 |
| MBLAC2 | -2.01 | 1.13E-07 | 5.48E-06 |
| MBNL2 | -1.93 | 2.00E-06 | 5.53E-05 |
| MCM6 | -1.50 | 1.02E-05 | 1.92E-04 |
| MEGF9 | -1.70 | 3.43E-05 | 4.93E-04 |
| METTL21A | -1.77 | 1.46E-05 | 2.55E-04 |
| MFNG | -1.53 | 4.43E-06 | 1.01E-04 |
| MGC12916 | 2.71 | 2.98E-08 | 1.85E-06 |
| MITF | -1.57 | 2.62E-10 | 4.02E-08 |
| MKL2 | -2.41 | 1.11E-07 | 5.43E-06 |
| MLKL | 2.07 | 1.68E-09 | 1.88E-07 |
| MOB3B | 2.05 | 1.61E-08 | 1.13E-06 |
| MRPL35 | -1.74 | 6.68E-08 | 3.62E-06 |
| MVB12A | 1.64 | 5.70E-08 | 3.22E-06 |
| MYC | -1.92 | 6.54E-04 | 4.82E-03 |
| N4BP1 | 1.76 | 8.81E-08 | 4.53E-06 |
| N4BP2L1 | 3.02 | 2.65E-12 | 8.24E-10 |
| NAA40 | -2.11 | 1.73E-10 | 2.89E-08 |
| NCOA7 | 2.63 | 1.77E-10 | 2.91E-08 |
| NCR3LG1 | 1.68 | 6.73E-05 | 8.36E-04 |
| NCS1 | 1.56 | 1.18E-08 | 8.72E-07 |
| NEDD4L | 2.09 | 1.09E-04 | 1.21E-03 |
| NEDD9 | -1.93 | 4.14E-06 | 9.66E-05 |
| NFATC1 | -1.71 | 5.04E-06 | 1.12E-04 |
| NIN | -1.59 | 1.96E-06 | 5.45E-05 |
| NKX3-1 | 4.94 | 8.47E-10 | 1.03E-07 |
| NLRC4 | -2.04 | 2.33E-05 | 3.62E-04 |
| NOD2 | 1.94 | 1.54E-03 | 9.33E-03 |
| NR1D2 | -1.93 | 2.93E-04 | 2.57E-03 |
| NR4A3 | 3.15 | 8.64E-06 | 1.68E-04 |
| NRP2 | 1.67 | 4.20E-07 | 1.60E-05 |
| NT5C3A | 3.12 | 2.64E-11 | 5.67E-09 |
| NT5DC1 | -1.57 | 6.48E-06 | 1.35E-04 |
| NUB1 | 1.58 | 3.59E-07 | 1.41E-05 |
| NUDT7 | -2.02 | 3.16E-04 | 2.73E-03 |
| NUPR1 | 2.48 | 1.03E-03 | 6.82E-03 |
| OGFR | 1.89 | 3.16E-10 | 4.76E-08 |
| OMA1 | -1.74 | 2.33E-05 | 3.62E-04 |
| OPTN | 1.95 | 4.46E-06 | 1.02E-04 |
| OR52K3P | 1.92 | 1.66E-06 | 4.74E-05 |
| OSGEPL1 | -2.31 | 1.50E-06 | 4.39E-05 |
| P2RX7 | 1.64 | 3.22E-04 | 2.77E-03 |
| PAK1 | -1.65 | 6.14E-07 | 2.14E-05 |
| PANX1 | 2.52 | 9.30E-13 | 3.58E-10 |
| PARD6B | -1.64 | 1.15E-03 | 7.45E-03 |
| PARP10 | 1.98 | 6.74E-11 | 1.24E-08 |
| PARP11 | 1.54 | 7.74E-10 | 9.61E-08 |
| PARP12 | 1.62 | 5.31E-11 | 1.04E-08 |
| PARP14 | 3.31 | 6.05E-15 | 7.97E-12 |
| PAX5 | 1.75 | 4.55E-06 | 1.03E-04 |
| PCMTD2 | -1.57 | 1.39E-05 | 2.46E-04 |
| PDE4DIP | 1.61 | 5.39E-04 | 4.13E-03 |
| PER2 | -1.82 | 9.63E-06 | 1.84E-04 |
| PGAP1 | 2.21 | 4.43E-07 | 1.67E-05 |
| PHACTR4 | 2.25 | 8.04E-10 | 9.84E-08 |
| PHF11 | 1.78 | 1.34E-09 | 1.54E-07 |
| PIGM | -1.52 | 5.98E-06 | 1.28E-04 |
| PLD1 | 1.69 | 6.59E-04 | 4.85E-03 |
| PMAIP1 | 2.52 | 1.04E-08 | 7.93E-07 |
| PMEPA1 | 2.26 | 1.43E-05 | 2.51E-04 |
| PNPT1 | 2.52 | 7.80E-08 | 4.12E-06 |
| POP5 | -1.56 | 1.11E-03 | 7.25E-03 |
| PPARGC1B | -1.91 | 2.77E-08 | 1.75E-06 |
| PPAT | -1.61 | 9.51E-07 | 3.06E-05 |
| PPM1K | 3.97 | 2.21E-14 | 1.69E-11 |
| PPP1R3B | 2.72 | 5.49E-08 | 3.11E-06 |
| PPP3CA | -1.69 | 2.57E-10 | 4.00E-08 |
| PRAM1 | -2.42 | 3.10E-07 | 1.25E-05 |
| PRKAR2B | -1.51 | 1.52E-03 | 9.22E-03 |
| PSIP1 | -1.89 | 1.61E-06 | 4.64E-05 |
| PSPH | -1.67 | 8.62E-04 | 5.94E-03 |
| PTPN22 | -1.99 | 5.31E-05 | 6.93E-04 |
| PTX3 | 3.40 | 3.00E-06 | 7.48E-05 |
| PVR | 1.79 | 1.32E-05 | 2.35E-04 |
| PVRL2 | 2.04 | 7.20E-12 | 1.90E-09 |
| PXK | -1.69 | 2.13E-07 | 9.04E-06 |
| RAB11FIP1 | -1.93 | 7.49E-08 | 3.99E-06 |
| RAB24 | 1.54 | 9.95E-06 | 1.88E-04 |
| RAB8B | 1.52 | 4.96E-11 | 9.88E-09 |
| RAD9A | 1.62 | 3.25E-07 | 1.30E-05 |
| RASSF2 | -2.82 | 3.29E-09 | 3.22E-07 |
| RBBP6 | 1.58 | 6.28E-12 | 1.72E-09 |
| RBL1 | -1.65 | 2.47E-08 | 1.59E-06 |
| RCBTB2 | -1.80 | 4.32E-06 | 9.93E-05 |
| REL | 1.55 | 1.49E-07 | 6.82E-06 |
| RHOBTB3 | 1.76 | 5.35E-07 | 1.93E-05 |
| RHOU | 2.18 | 2.78E-06 | 7.07E-05 |
| RMDN2 | -1.72 | 1.96E-05 | 3.17E-04 |
| RNF141 | -1.62 | 4.88E-04 | 3.83E-03 |
| RNF144A | 1.64 | 2.81E-06 | 7.13E-05 |
| RNF166 | -2.09 | 4.82E-08 | 2.76E-06 |
| RNF44 | -1.83 | 1.56E-08 | 1.10E-06 |
| RUNX3 | 2.31 | 2.39E-06 | 6.37E-05 |
| SAV1 | 1.97 | 1.29E-09 | 1.49E-07 |
| SCO2 | 1.66 | 6.98E-09 | 5.81E-07 |
| SDE2 | 1.70 | 1.88E-06 | 5.27E-05 |
| SEPSECS | -1.86 | 6.18E-10 | 7.95E-08 |
| SERPINE2 | 2.45 | 5.97E-07 | 2.09E-05 |
| SH2D3C | -2.27 | 1.51E-07 | 6.87E-06 |
| SKA2 | -1.93 | 1.34E-05 | 2.38E-04 |
| SLAMF7 | 1.70 | 3.57E-05 | 5.08E-04 |
| SLC25A28 | 2.69 | 2.28E-12 | 7.25E-10 |
| SLCO4A1 | 2.15 | 7.03E-06 | 1.43E-04 |
| SLFN12 | 1.61 | 2.61E-05 | 3.96E-04 |
| SLFN5 | 2.79 | 3.90E-05 | 5.42E-04 |
| SLITRK4 | -2.31 | 8.19E-04 | 5.73E-03 |
| SMARCA2 | -1.64 | 2.38E-10 | 3.80E-08 |
| SNHG19 | -1.62 | 2.48E-04 | 2.27E-03 |
| SNX30 | -1.91 | 2.92E-06 | 7.32E-05 |
| SP100 | 1.96 | 2.20E-12 | 7.25E-10 |
| SP110 | 4.07 | 2.12E-13 | 1.23E-10 |
| SP140L | 1.54 | 1.54E-08 | 1.09E-06 |
| SPATS2 | -1.96 | 7.79E-07 | 2.58E-05 |
| SPATS2L | 1.61 | 9.91E-06 | 1.88E-04 |
| SPTLC2 | 1.55 | 8.94E-08 | 4.57E-06 |
| SRGAP1 | 1.67 | 1.30E-06 | 3.94E-05 |
| SRGAP2C | 1.52 | 8.95E-06 | 1.73E-04 |
| SSH2 | -1.81 | 1.63E-05 | 2.76E-04 |
| ST6GALNAC2 | 2.22 | 3.16E-04 | 2.73E-03 |
| STAMBPL1 | 1.60 | 7.96E-05 | 9.48E-04 |
| STAT3 | 1.82 | 8.67E-12 | 2.21E-09 |
| STAU2 | -1.87 | 5.82E-06 | 1.25E-04 |
| STOML1 | 2.05 | 5.15E-11 | 1.01E-08 |
| STXBP1 | -1.51 | 4.78E-05 | 6.39E-04 |
| SVIL | 1.91 | 5.22E-04 | 4.03E-03 |
| TBC1D10C | -2.10 | 1.85E-05 | 3.03E-04 |
| TBL1XR1 | -1.53 | 4.80E-13 | 2.10E-10 |
| TCHH | 1.92 | 1.50E-04 | 1.54E-03 |
| TDRD7 | 2.49 | 1.01E-13 | 6.28E-11 |
| TGFA | 3.32 | 3.91E-04 | 3.23E-03 |
| TGFBR2 | -2.30 | 9.65E-07 | 3.10E-05 |
| THAP2 | 2.67 | 1.48E-06 | 4.35E-05 |
| THYN1 | -1.78 | 5.17E-11 | 1.01E-08 |
| TIFAB | -2.66 | 1.27E-03 | 8.03E-03 |
| TJP1 | 1.91 | 3.45E-06 | 8.33E-05 |
| TMEM110 | 1.76 | 1.46E-05 | 2.55E-04 |
| TMEM117 | -2.12 | 4.21E-06 | 9.79E-05 |
| TMEM140 | 2.66 | 3.73E-07 | 1.46E-05 |
| TMEM217 | 3.14 | 1.33E-07 | 6.28E-06 |
| TNFAIP2 | 1.72 | 7.59E-05 | 9.17E-04 |
| TNFRSF10A | 1.57 | 1.00E-05 | 1.89E-04 |
| TNFRSF9 | 2.82 | 2.26E-08 | 1.48E-06 |
| TNFSF9 | 3.20 | 8.08E-09 | 6.56E-07 |
| TP53 | -1.88 | 4.61E-07 | 1.72E-05 |
| TPBG | 1.50 | 9.33E-04 | 6.31E-03 |
| TRAFD1 | 1.95 | 7.27E-13 | 2.92E-10 |
| TRANK1 | 2.06 | 1.69E-05 | 2.84E-04 |
| TRIM14 | 1.58 | 4.45E-07 | 1.67E-05 |
| TRIM21 | 1.79 | 4.19E-13 | 1.97E-10 |
| TRIM22 | 2.77 | 7.37E-08 | 3.94E-06 |
| TRIM25 | 3.20 | 5.36E-14 | 3.75E-11 |
| TRIM26 | 1.65 | 6.82E-14 | 4.54E-11 |
| TRIM5 | 2.26 | 1.05E-08 | 7.98E-07 |
| TRIM56 | 1.70 | 2.47E-10 | 3.90E-08 |
| TSHZ1 | -1.88 | 3.98E-05 | 5.51E-04 |
| TSLP | 3.01 | 1.65E-04 | 1.66E-03 |
| TTC8 | -1.86 | 9.10E-09 | 7.21E-07 |
| UBE2L6 | 1.69 | 4.35E-12 | 1.24E-09 |
| USP2 | -2.03 | 2.02E-04 | 1.93E-03 |
| USP42 | 1.62 | 3.96E-09 | 3.71E-07 |
| UTRN | -1.94 | 1.18E-09 | 1.39E-07 |
| VWA5A | -1.82 | 9.09E-05 | 1.05E-03 |
| WDR91 | -1.76 | 2.57E-04 | 2.33E-03 |
| XIRP1 | 3.04 | 8.88E-06 | 1.72E-04 |
| YPEL4 | -2.24 | 2.02E-06 | 5.57E-05 |
| ZADH2 | -1.69 | 3.37E-06 | 8.22E-05 |
| ZBP1 | 2.37 | 1.08E-04 | 1.20E-03 |
| ZBTB21 | -1.57 | 1.99E-06 | 5.52E-05 |
| ZBTB46 | -1.73 | 1.16E-04 | 1.27E-03 |
| ZDHHC9 | 1.98 | 5.69E-04 | 4.32E-03 |
| ZEB2 | -1.75 | 6.06E-10 | 7.88E-08 |
| ZFP36 | 1.89 | 1.61E-07 | 7.21E-06 |
| ZFP36L1 | -1.54 | 4.86E-04 | 3.82E-03 |
| ZNF107 | 1.66 | 7.56E-05 | 9.13E-04 |
| ZNF362 | -1.65 | 8.87E-08 | 4.55E-06 |
| ZNF780A | -1.57 | 1.01E-04 | 1.14E-03 |
