# Supplementary material for: Essential genes of the macrophage response to Staphylococcus aureus exposure
Source: Cell Mol Biol Lett. 2018 May 23;23:25. doi: 10.1186/s11658-018-0090-4 (PMC5966896; doi:10.1186/s11658-018-0090-4)
Supplement: Supplementary file 3 — Table S3. Two hundred twenty-seven DEGs obtained in Comparison 2 (|log2(FC)| > 1.5 & adjusted p < 0.01). (DOCX 25 kb) [file 11658_2018_90_MOESM3_ESM.docx]

**Table S3. 227 DEGs obtained in Comparison 2 (|log2(FC)| > 1.5 & adjusted p< 0.01)**

| Gene_symbol | LogFC | P value | Adjusted p value |
| --- | --- | --- | --- |
| ABAT | -1.76 | 2.69E-04 | 3.05E-03 |
| ABCC4 | -1.61 | 2.24E-05 | 4.75E-04 |
| ABHD2 | -1.81 | 1.46E-06 | 6.04E-05 |
| ABI3 | -1.80 | 7.42E-09 | 1.42E-06 |
| ACACA | -1.95 | 1.82E-06 | 7.07E-05 |
| ACAT2 | -1.79 | 1.20E-03 | 9.17E-03 |
| ACSF2 | -1.95 | 4.94E-05 | 8.48E-04 |
| ACSL5 | 1.59 | 1.77E-07 | 1.33E-05 |
| ADD3 | -1.60 | 1.86E-04 | 2.31E-03 |
| ADK | -1.51 | 1.06E-03 | 8.31E-03 |
| ADORA2B | -1.63 | 9.80E-09 | 1.66E-06 |
| AGPAT5 | -1.84 | 1.53E-04 | 1.98E-03 |
| AGPS | -2.06 | 9.04E-06 | 2.42E-04 |
| AIFM2 | 1.78 | 1.07E-07 | 9.41E-06 |
| ALDH7A1 | -1.59 | 3.64E-04 | 3.79E-03 |
| AMDHD1 | -1.90 | 7.03E-04 | 6.15E-03 |
| AMICA1 | -1.96 | 1.73E-05 | 3.88E-04 |
| ANGPTL6 | -1.53 | 2.71E-05 | 5.50E-04 |
| APOL3 | 2.40 | 5.59E-05 | 9.31E-04 |
| AREG | 3.84 | 7.39E-04 | 6.37E-03 |
| ARHGEF9 | -1.56 | 8.35E-06 | 2.26E-04 |
| ARID3B | 2.10 | 2.30E-04 | 2.74E-03 |
| ARID5B | 1.73 | 7.96E-06 | 2.18E-04 |
| ARL10 | -1.75 | 3.60E-05 | 6.76E-04 |
| ASAP2 | 1.75 | 1.05E-06 | 4.75E-05 |
| ASB1 | -1.54 | 8.02E-06 | 2.19E-04 |
| ASB2 | -1.63 | 1.27E-03 | 9.52E-03 |
| ATP1B4 | -2.70 | 2.80E-04 | 3.15E-03 |
| ATP8A1 | -2.06 | 1.34E-05 | 3.23E-04 |
| BRI3BP | -1.62 | 1.00E-07 | 9.03E-06 |
| BST2 | 1.52 | 2.34E-07 | 1.65E-05 |
| C11orf21 | -2.59 | 7.37E-04 | 6.37E-03 |
| C11orf45 | -2.89 | 1.46E-07 | 1.18E-05 |
| C1orf95 | -1.68 | 2.51E-04 | 2.91E-03 |
| C2 | 2.05 | 2.41E-04 | 2.82E-03 |
| C20orf27 | -2.03 | 3.08E-05 | 6.02E-04 |
| C22orf39 | -1.59 | 1.25E-06 | 5.37E-05 |
| C2orf76 | -1.92 | 9.22E-06 | 2.45E-04 |
| CADM1 | -2.57 | 2.24E-05 | 4.75E-04 |
| CAMK2D | -1.69 | 4.28E-06 | 1.37E-04 |
| CARD16 | 2.59 | 2.99E-05 | 5.90E-04 |
| CASP4 | 1.57 | 9.47E-07 | 4.42E-05 |
| CBX5 | -2.07 | 1.08E-06 | 4.84E-05 |
| CCDC50 | 1.64 | 9.62E-06 | 2.53E-04 |
| CCNB2 | -2.44 | 5.61E-04 | 5.20E-03 |
| CD180 | -2.53 | 3.18E-05 | 6.15E-04 |
| CD1E | -2.41 | 1.14E-04 | 1.59E-03 |
| CD36 | -3.55 | 5.16E-04 | 4.89E-03 |
| CD59 | -1.61 | 3.42E-04 | 3.61E-03 |
| CD93 | 3.42 | 6.60E-04 | 5.87E-03 |
| CDK5RAP2 | 1.51 | 7.71E-04 | 6.57E-03 |
| CENPU | -2.09 | 9.59E-04 | 7.72E-03 |
| CHI3L2 | 2.13 | 6.66E-04 | 5.90E-03 |
| CKAP2 | -1.82 | 1.68E-05 | 3.78E-04 |
| CTSLP8 | 2.20 | 2.62E-07 | 1.78E-05 |
| CXorf57 | -1.68 | 2.88E-05 | 5.72E-04 |
| CYSLTR1 | -1.64 | 1.01E-03 | 8.01E-03 |
| DANCR | -2.13 | 1.07E-06 | 4.81E-05 |
| DCUN1D3 | 2.09 | 9.00E-08 | 8.51E-06 |
| DHFR | -1.86 | 3.96E-04 | 4.04E-03 |
| DNAJC21 | -1.54 | 4.91E-06 | 1.53E-04 |
| DNPH1 | -1.53 | 6.68E-05 | 1.06E-03 |
| DPEP2 | -2.87 | 1.47E-05 | 3.46E-04 |
| DSP | -1.53 | 9.55E-04 | 7.71E-03 |
| EBPL | -2.04 | 7.62E-04 | 6.51E-03 |
| EDN1 | 5.32 | 9.30E-07 | 4.38E-05 |
| EEPD1 | -2.45 | 1.97E-05 | 4.29E-04 |
| EHD3 | -2.20 | 4.91E-06 | 1.53E-04 |
| EMILIN1 | 1.88 | 1.13E-03 | 8.71E-03 |
| ENTPD7 | 1.87 | 1.31E-05 | 3.17E-04 |
| EPHB6 | -1.78 | 5.64E-08 | 6.11E-06 |
| ERMP1 | -1.84 | 1.21E-04 | 1.67E-03 |
| ETV5 | -1.61 | 7.82E-05 | 1.20E-03 |
| FA2H | -2.55 | 4.33E-04 | 4.32E-03 |
| FAM129A | 1.55 | 1.41E-04 | 1.88E-03 |
| FAM213B | -1.74 | 4.33E-04 | 4.32E-03 |
| FAM49B | -2.03 | 1.85E-04 | 2.31E-03 |
| FCGR2B | 3.31 | 6.41E-04 | 5.73E-03 |
| FHL1 | -3.68 | 3.97E-05 | 7.26E-04 |
| FITM2 | -1.74 | 9.46E-07 | 4.42E-05 |
| FOSL1 | 1.89 | 1.52E-05 | 3.53E-04 |
| FPR1 | 2.38 | 2.24E-04 | 2.69E-03 |
| FXN | -1.53 | 7.08E-05 | 1.11E-03 |
| GALM | -1.87 | 9.78E-06 | 2.55E-04 |
| GIMAP8 | 1.55 | 1.26E-05 | 3.09E-04 |
| GINS1 | -2.07 | 2.37E-06 | 8.62E-05 |
| GLCCI1 | -1.93 | 2.39E-04 | 2.81E-03 |
| GLIS3 | 2.77 | 5.51E-06 | 1.64E-04 |
| GNA15 | 1.67 | 1.13E-10 | 7.73E-08 |
| GPAM | -1.55 | 7.74E-06 | 2.13E-04 |
| GPD1L | -2.45 | 6.02E-05 | 9.87E-04 |
| GRAMD3 | 1.88 | 2.23E-05 | 4.73E-04 |
| H2AFY | -1.57 | 7.37E-08 | 7.37E-06 |
| HADH | -2.42 | 2.03E-06 | 7.72E-05 |
| HBEGF | 2.72 | 2.82E-04 | 3.16E-03 |
| HCG11 | -1.72 | 2.30E-06 | 8.41E-05 |
| HILPDA | -1.51 | 4.78E-05 | 8.27E-04 |
| HNRNPDL | -1.53 | 2.39E-04 | 2.81E-03 |
| HPS3 | 1.80 | 1.43E-10 | 9.07E-08 |
| HPSE | 3.14 | 3.97E-04 | 4.04E-03 |
| IL31RA | 4.13 | 2.80E-05 | 5.61E-04 |
| IL32 | 3.88 | 1.23E-03 | 9.29E-03 |
| IL4I1 | 1.52 | 3.00E-04 | 3.30E-03 |
| IMPA2 | -2.68 | 5.11E-05 | 8.71E-04 |
| ITGB5 | -1.84 | 4.71E-05 | 8.20E-04 |
| ITPKB | -1.91 | 5.25E-06 | 1.59E-04 |
| JAG1 | 1.80 | 5.42E-04 | 5.07E-03 |
| KCNJ1 | -2.79 | 7.47E-04 | 6.43E-03 |
| KCTD15 | -1.74 | 2.03E-05 | 4.40E-04 |
| KDM7A | 1.78 | 1.88E-04 | 2.34E-03 |
| KIAA0930 | -1.88 | 4.18E-07 | 2.40E-05 |
| KLF11 | -1.54 | 1.34E-03 | 9.93E-03 |
| LAD1 | 1.54 | 1.22E-03 | 9.24E-03 |
| LAMP2 | -1.74 | 8.55E-04 | 7.09E-03 |
| LAMTOR3 | -1.67 | 4.01E-06 | 1.31E-04 |
| LDLRAD4 | -1.87 | 3.65E-04 | 3.80E-03 |
| LGALS3BP | 2.50 | 5.99E-05 | 9.83E-04 |
| LINC01010 | -2.07 | 1.50E-05 | 3.50E-04 |
| LINC01093 | 1.86 | 2.20E-05 | 4.69E-04 |
| LINC01503 | -2.03 | 1.54E-06 | 6.28E-05 |
| LOC285181 | -1.78 | 8.49E-04 | 7.05E-03 |
| LRP12 | 1.81 | 7.31E-07 | 3.67E-05 |
| LTA4H | -1.75 | 2.61E-07 | 1.78E-05 |
| M1AP | -1.64 | 2.42E-04 | 2.84E-03 |
| MAD2L1 | -2.28 | 3.96E-04 | 4.04E-03 |
| MAP3K9 | 1.74 | 5.86E-04 | 5.36E-03 |
| MAPRE2 | -1.97 | 1.80E-04 | 2.26E-03 |
| MARCKS | 1.99 | 7.09E-04 | 6.19E-03 |
| MCM2 | -1.73 | 1.47E-04 | 1.94E-03 |
| MCM7 | -1.54 | 1.03E-03 | 8.15E-03 |
| MMP3 | 4.46 | 5.72E-04 | 5.27E-03 |
| MS4A14 | 1.62 | 2.91E-05 | 5.77E-04 |
| MT1F | 1.91 | 1.74E-06 | 6.85E-05 |
| MT1G | 1.91 | 5.12E-06 | 1.57E-04 |
| MT1H | 1.80 | 4.09E-06 | 1.32E-04 |
| MT1HL1 | 1.57 | 4.19E-06 | 1.35E-04 |
| MT1M | 3.30 | 5.77E-04 | 5.29E-03 |
| MT1X | 1.96 | 6.71E-07 | 3.49E-05 |
| MT2A | 1.83 | 1.92E-07 | 1.41E-05 |
| MTR | -1.54 | 1.95E-07 | 1.43E-05 |
| NCAPG2 | -1.72 | 5.86E-05 | 9.68E-04 |
| NDST1 | 1.65 | 5.08E-08 | 5.64E-06 |
| NFKBIZ | 1.88 | 6.32E-04 | 5.67E-03 |
| NINL | -2.28 | 1.56E-05 | 3.60E-04 |
| NLN | -1.59 | 2.25E-06 | 8.27E-05 |
| NLRP3 | 2.26 | 9.50E-06 | 2.51E-04 |
| NRGN | -2.53 | 2.78E-05 | 5.58E-04 |
| NUSAP1 | -1.77 | 4.40E-04 | 4.35E-03 |
| OSBPL3 | -1.94 | 1.06E-04 | 1.50E-03 |
| PAQR8 | -2.42 | 5.93E-05 | 9.77E-04 |
| PCYOX1L | -1.69 | 1.68E-04 | 2.14E-03 |
| PFKFB2 | -1.63 | 1.70E-07 | 1.29E-05 |
| PINLYP | 1.89 | 8.55E-04 | 7.09E-03 |
| PLA2G12A | -1.62 | 5.82E-04 | 5.33E-03 |
| PLEKHG2 | 1.59 | 1.96E-04 | 2.41E-03 |
| PLEKHM3 | 1.96 | 5.96E-05 | 9.81E-04 |
| PNPLA1 | 2.24 | 2.07E-04 | 2.52E-03 |
| PNRC1 | 1.64 | 1.55E-04 | 2.01E-03 |
| PPP3CC | 1.66 | 1.84E-08 | 2.65E-06 |
| PRKCA | -1.51 | 5.45E-04 | 5.09E-03 |
| PSMB9 | 1.59 | 8.70E-11 | 6.25E-08 |
| PSME2 | 1.56 | 1.94E-13 | 7.31E-10 |
| PTGS1 | -1.52 | 9.72E-04 | 7.79E-03 |
| RAB11A | -1.55 | 6.53E-07 | 3.41E-05 |
| RAB11FIP4 | -1.78 | 3.16E-05 | 6.13E-04 |
| RAD51AP1 | -1.86 | 4.91E-06 | 1.53E-04 |
| RAP2B | -2.27 | 2.99E-05 | 5.90E-04 |
| RGS20 | -2.39 | 9.87E-04 | 7.87E-03 |
| RHOBTB1 | -1.90 | 4.22E-04 | 4.24E-03 |
| RNF128 | -2.59 | 7.82E-05 | 1.20E-03 |
| RTN4R | -1.83 | 3.66E-06 | 1.22E-04 |
| SAMD4A | -2.38 | 2.45E-06 | 8.86E-05 |
| SAMSN1 | 2.47 | 4.78E-05 | 8.27E-04 |
| SAP30L | -2.09 | 2.59E-04 | 2.97E-03 |
| SCARB1 | -2.22 | 3.75E-04 | 3.89E-03 |
| 11-Sep | -1.57 | 4.65E-04 | 4.51E-03 |
| SERPIND1 | 2.60 | 3.10E-05 | 6.05E-04 |
| SERPINF1 | -2.61 | 9.28E-05 | 1.36E-03 |
| SESN2 | 2.02 | 1.36E-04 | 1.83E-03 |
| SIK3 | 1.87 | 7.71E-09 | 1.44E-06 |
| SLC12A2 | -1.83 | 6.64E-06 | 1.90E-04 |
| SLC16A1 | -1.70 | 3.38E-05 | 6.44E-04 |
| SLC25A29 | -1.93 | 2.76E-11 | 2.70E-08 |
| SLC29A1 | -1.70 | 1.39E-04 | 1.86E-03 |
| SLC39A8 | 1.83 | 2.55E-05 | 5.26E-04 |
| SLC43A2 | 1.80 | 7.64E-07 | 3.76E-05 |
| SLC46A3 | -2.34 | 3.35E-05 | 6.39E-04 |
| SLCO2B1 | -1.94 | 6.81E-04 | 6.00E-03 |
| SLCO4C1 | -2.14 | 4.72E-05 | 8.22E-04 |
| SNRNP25 | -1.96 | 1.72E-05 | 3.86E-04 |
| SOCS2 | 1.52 | 9.94E-04 | 7.92E-03 |
| SORD | -1.51 | 4.08E-05 | 7.40E-04 |
| SP140 | 2.93 | 7.10E-11 | 5.34E-08 |
| SPARC | -1.81 | 9.20E-07 | 4.34E-05 |
| SSR1 | -1.93 | 1.03E-08 | 1.72E-06 |
| STAB1 | -3.33 | 1.22E-03 | 9.24E-03 |
| STAP2 | 1.58 | 2.51E-06 | 8.99E-05 |
| STK17A | 1.89 | 1.72E-06 | 6.79E-05 |
| SYNJ2BP | -1.55 | 9.60E-07 | 4.46E-05 |
| TECR | -1.55 | 4.02E-08 | 4.85E-06 |
| THEM4 | -1.88 | 5.91E-04 | 5.40E-03 |
| TLR5 | -2.22 | 6.39E-04 | 5.72E-03 |
| TMC8 | -1.77 | 6.49E-04 | 5.79E-03 |
| TMEM173 | 2.90 | 1.87E-09 | 5.37E-07 |
| TMEM97 | -3.67 | 1.35E-05 | 3.24E-04 |
| TNFRSF11A | -2.28 | 5.67E-04 | 5.23E-03 |
| TNFRSF21 | -1.78 | 4.15E-05 | 7.50E-04 |
| TNFRSF4 | 2.31 | 6.22E-04 | 5.61E-03 |
| TPCN1 | -2.16 | 9.76E-08 | 8.88E-06 |
| TRIM37 | -2.53 | 4.63E-05 | 8.12E-04 |
| TTF2 | -1.72 | 1.20E-07 | 1.02E-05 |
| TTK | -2.05 | 1.21E-03 | 9.23E-03 |
| TXNDC16 | -1.98 | 4.42E-04 | 4.37E-03 |
| ULK2 | 1.67 | 8.06E-05 | 1.22E-03 |
| USP31 | -1.63 | 4.57E-06 | 1.45E-04 |
| USP46 | -1.53 | 1.17E-06 | 5.08E-05 |
| VAPB | -1.68 | 3.18E-06 | 1.09E-04 |
| VSIG4 | -2.12 | 8.92E-04 | 7.31E-03 |
| XPNPEP3 | -1.58 | 3.31E-07 | 2.07E-05 |
| XPOT | -1.63 | 1.07E-05 | 2.72E-04 |
| XYLT1 | -2.02 | 8.13E-04 | 6.83E-03 |
| ZBED3 | -1.86 | 2.11E-04 | 2.56E-03 |
| ZBTB44 | -1.63 | 5.50E-05 | 9.20E-04 |
| ZBTB8A | -1.86 | 9.00E-05 | 1.33E-03 |
| ZMAT3 | -1.57 | 1.03E-06 | 4.68E-05 |
| ZMYND15 | 1.55 | 9.35E-06 | 2.47E-04 |
| ZNF677 | -1.95 | 2.39E-04 | 2.81E-03 |
