# Supplementary material for: Essential genes of the macrophage response to Staphylococcus aureus exposure
Source: Cell Mol Biol Lett. 2018 May 23;23:25. doi: 10.1186/s11658-018-0090-4 (PMC5966896; doi:10.1186/s11658-018-0090-4)
Supplement: Supplementary file 4 — Table S4. Fifty-one DEGs obtained in Comparison 3 (|log2(fold change)| > 1.5 & adjusted p value < 0.01). (DOCX 17 kb) [file 11658_2018_90_MOESM4_ESM.docx]

**Table S4. 51 DEGs obtained in comparison 3 (|log2(fold change)| > 1.5 & adjusted p value < 0.01)**

| Gene_symbol | LogFC | P. value | Adjusted p value |
| --- | --- | --- | --- |
| ACAT1 | -1.52 | 5.47E-05 | 2.37E-03 |
| BTG1 | 1.61 | 1.80E-05 | 1.16E-03 |
| C16orf45 | 1.95 | 2.76E-05 | 1.55E-03 |
| C1RL | 1.87 | 2.30E-04 | 6.15E-03 |
| C22orf42 | 3.14 | 6.88E-09 | 7.23E-06 |
| CDCP1 | 1.87 | 7.72E-05 | 2.99E-03 |
| CHST2 | 1.75 | 3.52E-04 | 8.40E-03 |
| COL7A1 | 1.85 | 3.84E-06 | 4.05E-04 |
| CPVL | -2.56 | 2.53E-04 | 6.58E-03 |
| CRTAM | -2.59 | 1.84E-04 | 5.25E-03 |
| CYBRD1 | -1.56 | 3.22E-04 | 7.88E-03 |
| DDAH2 | -1.64 | 8.96E-07 | 1.97E-04 |
| DGAT2 | 2.33 | 4.02E-05 | 1.97E-03 |
| EBF1 | 2.57 | 1.43E-04 | 4.55E-03 |
| FABP3 | -2.60 | 1.61E-04 | 4.84E-03 |
| GPR64 | 3.51 | 4.69E-05 | 2.14E-03 |
| HOPX | 1.74 | 2.70E-05 | 1.53E-03 |
| HPGDS | -3.22 | 9.13E-05 | 3.33E-03 |
| ITGA7 | 2.63 | 5.51E-05 | 2.39E-03 |
| ITGB7 | 2.45 | 1.15E-04 | 3.91E-03 |
| ITPR3 | 2.56 | 5.53E-10 | 1.13E-06 |
| LAT | 1.58 | 1.28E-05 | 9.34E-04 |
| LOC728084 | 1.74 | 1.45E-04 | 4.57E-03 |
| LY86 | -1.76 | 2.58E-04 | 6.66E-03 |
| MARCKSL1 | 2.34 | 8.25E-05 | 3.12E-03 |
| MDGA1 | 1.70 | 4.61E-08 | 2.93E-05 |
| METTL7B | 2.69 | 1.59E-04 | 4.83E-03 |
| MGAM | 1.97 | 2.06E-04 | 5.70E-03 |
| MICALCL | 2.01 | 6.29E-06 | 5.80E-04 |
| MNDA | -3.22 | 3.95E-04 | 9.11E-03 |
| MSC | 1.59 | 1.80E-04 | 5.19E-03 |
| NCAM1 | 3.11 | 1.58E-06 | 2.48E-04 |
| NDRG2 | 2.18 | 5.23E-05 | 2.31E-03 |
| ORM1 | 2.30 | 2.45E-04 | 6.43E-03 |
| RASL12 | 1.91 | 3.02E-07 | 9.95E-05 |
| RHBDD2 | 2.00 | 3.27E-06 | 3.68E-04 |
| RRAD | 3.17 | 5.25E-05 | 2.32E-03 |
| SERPINE1 | 1.75 | 4.38E-04 | 9.82E-03 |
| SLC24A3 | 2.40 | 2.14E-05 | 1.30E-03 |
| SLC51B | 2.70 | 9.18E-06 | 7.24E-04 |
| SORBS1 | 2.66 | 1.61E-04 | 4.84E-03 |
| SPATA12 | -1.57 | 1.92E-04 | 5.40E-03 |
| STMN1 | -1.59 | 4.13E-04 | 9.44E-03 |
| TAGLN3 | 2.58 | 1.42E-06 | 2.38E-04 |
| TBL1X | 2.10 | 9.56E-06 | 7.48E-04 |
| TBX21 | 1.58 | 3.37E-04 | 8.12E-03 |
| TLE1 | 1.91 | 8.56E-08 | 4.33E-05 |
| TMEM37 | -2.05 | 2.85E-04 | 7.18E-03 |
| TNFSF8 | -3.36 | 1.81E-04 | 5.19E-03 |
| TRIM32 | -1.73 | 3.18E-05 | 1.69E-03 |
| TUBB2A | -1.97 | 4.37E-05 | 2.06E-03 |
